# Long Non-Coding RNAs Contribute to Glucose Starvation-Induced Dedifferentiation in Lung Adenocarcinoma

**DOI:** 10.3390/biom15111493

**Published:** 2025-10-23

**Authors:** Aparamita Pandey, Pasquale Saggese, Adriana Soto, Estefany Gomez, Martín Alcaraz, Claudio Scafoglio

**Affiliations:** 1 Division of Pulmonary Medicine, David Geffen School of Medicine and Jonsson Comprehensive Cancer Center, University of California Los Angeles, Los Angeles, CA 90095, USA; apandey@mednet.ucla.edu (A.P.); pasquale.saggese@uniroma1.it (P.S.); asoto07@g.ucla.edu (A.S.); stefgbruin24@g.ucla.edu (E.G.);; 2 Department of Biology and Biotechnologies Charles Darwin, University of Rome “Sapienza”, Piazzale Aldo Moro 5, 00185 Rome, Italy

**Keywords:** epitranscriptomics, glucose starvation, dedifferentiation, lung adenocarcinoma, long noncoding RNA, FTO, EZH2

## Abstract

Nutrient deprivation causes dedifferentiation in solid tumors, driving an aggressive phenotype. We previously showed that glucose starvation-induced dedifferentiation is driven by epigenetic changes induced by a deficit of alpha-ketoglutarate (α-KG). Deficient activity of α-KG-dependent histone demethylases leads to unbalanced hypermethylation of histone 3 on lysine 27 (H3K27) by methyltransferase EZH2. H3K27 hypermethylation is a key mechanism of starvation-induced dedifferentiation. Here, we investigate a new aspect of this mechanism and show that epitranscriptomic changes are also induced by glucose restriction. Specifically, hypermethylation of select long non-coding RNAs leads to their upregulation under glucose deprivation as a consequence of reduced activity of the RNA demethylase FTO. We identified LINC00662 as an lncRNA required for EZH2 recruitment to target gene promoters induced by low glucose. These findings characterize the epigenetic response to glucose restriction beyond histone methylation, revealing that RNA methylation of lncRNAs such as LINC00662 represents a parallel mechanism converging on EZH2.

## 1. Introduction

Lung cancer is the leading cause of cancer-related mortality worldwide [1]. Lung adenocarcinoma (LUAD) is the most prevalent type of lung cancer, accounting for ~50% of non-small cell lung cancers [2]. Despite advances in targeted therapies, LUAD remains difficult to treat, particularly in its advanced stages, where a loss of cellular differentiation is often observed [3]. One key driver of this loss of differentiation is glucose deprivation, which occurs when rapidly proliferating cancer cells outpace the development of an adequate blood supply [4]. In this context, metabolic reprogramming plays a critical role, and alpha-ketoglutarate (α-KG), a key metabolite in the tricarboxylic acid (TCA) cycle, emerges as an important regulator of cell differentiation in cancer. Glutamine deprivation drives dedifferentiation in the hypoxic core of solid tumors due to an α-KG deficit, leading to H3K27 hypermethylation and gene repression [5]. We previously showed that glucose deprivation also drives dedifferentiation and the transition to an aggressive phenotype in LUAD [4]. The role of α-KG in regulating cell differentiation is tightly tied to its role in regulating the activity of epigenetic enzymes [6,7,8,9]. In our previous study, we showed that glucose starvation causes a deficit in α-KG, leading to reduced activity of histone demethylases and hypermethylation of histone marks H3K27me3 and H3K4me3 [4]. We found that glucose deprivation increases the recruitment of histone H3K27 methyltransferase EZH2 on the promoter of *EGLN3*, a gene encoding for the prolyl hydroxylase that induces degradation of HIF1α. This leads to transcriptional repression of *EGLN3* and chronic activation of HIF1α even in normoxia (pseudohypoxia). HIF1α activation drives dedifferentiation, epithelial-to-mesenchymal transition, and acquisition of a more aggressive phenotype as measured by in vivo metastasis formation assay [4]. This phenotype could be mitigated by either EZH2 inhibition or HIF1α knockdown, pointing to the EZH2/EGLN3/HIF1α axis as a critical mediator of starvation-induced dedifferentiation [4].

Although histone demethylation is a critical regulator of cell differentiation, other enzymes are regulated by α-KG. Among these, RNA demethylases play an increasingly recognized role in cell differentiation and stemness in cancer [10,11,12]. N6-methyladenosine (m6A) is the most abundant epitranscriptomic modification and plays an important role in lung cancer development [13]. While several “writers” that add methyl groups to RNA adenosine have been described, only two “erasers” that can demethylate m6A—FTO and ALKBH5—have been identified [10]. Both FTO and ALKBH5 can act either as oncogenes or as tumor suppressors, according to the context and tissue of origin [14,15]. Long non-coding RNAs are emerging targets of m6A demethylation involved in cancer initiation and progression [10]. However, the role of RNA demethylases FTO and ALKBH5 in the epitranscriptomic response to glucose restriction is not known.

Here, we connect our previous findings on the role of EZH2 in starvation-induced dedifferentiation and increased aggressiveness with the novel discovery that the m6A demethylase FTO is required to maintain cell differentiation in LUAD cells. Deficient activity of FTO in glucose-starved cells leads to hypermethylation of some long non-coding RNAs, which promote EZH2 recruitment on the *EGLN3* promoter, promoting the engagement of the EZH2/EGLN3/HIF1α axis to drive dedifferentiation. This is important as EZH2 inhibitors are developed as new treatments for NSCLC, with several ongoing clinical trials (NCT05467748, NCT06644768, NCT06022757, NCT05023655). The detection of the dedifferentiation pathway in human LUADs could be used in the future as a predictive biomarker of response.

## 2. Materials and Methods

- Cell lines.

Human A549 and NCI-H358 (hereby called H358) cells were purchased from ATCC and maintained in Roswell Park Memorial Institute (RPMI) 1640 medium (Corning, Corning, NY, USA, #10-040CV) supplemented with 10% FBS and 5% penicillin–streptomycin. Cells were cultured in a humidified incubator at 37 °C and 5% CO_2_. All cells were tested for Mycoplasma after being received from ATCC and used within 10 passages.

- In Vitro Studies.

All experiments in cell lines were performed in biological triplicate. Cells were seeded at the same confluence and grown for 5 days in different glucose concentrations: physiological (5 mmol/L) and low (1 mmol/L). We used RPMI with glutamine and without glucose (Corning, #10-043CV), supplemented with 10% FBS, 1× penicillin–streptomycin and complemented with D-glucose (Gibco, Grand Island, NY, USA, #A2494001) to the desired final concentration, as indicated in the figures. All α-KG rescue experiments were conducted using 10 mmol/L dimethyl α-ketoglutarate (Sigma, Burlington, MA, USA, #13192-04-6) and α-Mannitol (Sigma, #M9647) as osmotic control.

- Small interfering RNA transfection.

For small interfering RNA (siRNA), knockdown of *LINC00662*, *CRNDE*, *FTO*, and *ALKBH5* was performed. A549 and H358 cells were cultured in either physiological (5 mmol/L) or low-glucose (1 mmol/L) conditions and transfected with 25 pmol/L of siRNAs for each target as follows: siRNAs for *FTO* and *ALKBH5* were transfected in physiological conditions, while siRNAs for *LINC00662* and *CRNDE* in low-glucose medium, using Lipofectamine RNAiMAX (Thermo Fisher Scientific, Waltham, MA, USA, #13778075). Scrambled siRNA was transfected as a negative control. Each target gene was silenced using two independent siRNAs to control for off-target effects. Scrambled siRNA was used at the same concentration (25 pmol/L) as the experimental siRNAs. Because the total incubation time in low glucose was 5 days, two separate transfections were performed on day 1 and on day 3.

On day 5, cells were collected and processed for RT-qPCR and Western blotting analysis for *FTO* and *ALKBH5*, and ChIP-qPCR for EZH2. Each siRNA was used individually. All siRNAs were purchased from QIAGEN (ordering information in Table S1).

- Total protein extraction.

For total protein extraction, cells were harvested, washed twice with ice-cold PBS-EDTA (0.5 mmol/L EDTA), lysed using RIPA buffer (50 mmol/L Tris-HCl pH 7.6, 150 mmol/L NaCl, 0.1% SDS, 0.5% sodium deoxycholate, 1% NP-40, 2 mm EDTA, 50 mmol/L NaF) for 15 min on ice, and centrifuged at 13,000 rpm for 30 min at +4 °C.

The resulting protein extracts were quantified using BCA Protein Assay (Thermo Fisher Scientific, #23225), followed by SDS-PAGE run and Western blotting analysis.

- Western blotting.

SDS-PAGE and Western blotting analysis were performed using standard protocols.

Western blot images were detected by iBright Imaging Systems (Invitrogen, Waltham, MA, USA). The protein bands detected were normalized to the β-actin antibody. Each immunodetection was derived from the same membrane and performed with the same exposure times according to the manufacturer’s antibody guidelines. Densitometry of the protein bands was performed with ImageJ (1.54g). Antibodies were purchased from CST (ordering information in Table S2).

- RNA extraction.

Total RNA was extracted from A549 and H358 by using TRI Reagent Solution (Applied Biosystem, Foster City, CA, USA, #AM9738), according to the manufacturer’s instructions. RNA concentration was assayed by NanoDrop 3300 Fluorospectrometer. Then, 1 μg of RNA was treated with DNase I (Thermo Fisher Scientific, #MAN0012000) and used for cDNA preparation.

- RT-qPCR.

cDNA was prepared using 1 μg of RNA with SensiFast RT Kit (Meridian Biosciences, Cincinnati, OH, USA, #BIO-65053). The SYBR green-based RT-PCR kit (Applied Biosystem, #A25742) was used using specific primers (see Supplementary Table S3). mRNA levels were normalized to *GAPDH* (ΔCt = Ct (gene of interest)—Ct (*GAPDH*)) and presented as relative mRNA expression (2ΔCt). All primers were designed by NCBI Primer-BLAST and purchased from Integrated DNA Technologies. The sequences of all primers used are presented in Table S3.

- RNA-seq, data analysis, and material availability.

To analyze the differential expression of the long non-coding RNAs, we used the dataset previously published from our laboratory (Array Express: E-MTAB-11253; Saggese et al., 2024 [4]). Differential expression was reported as |fold change| (FC) ≥ 1.5 along with associated adjusted *p* ≤ 0.05 computed according to Benjamini–Hochberg. Any additional information required to reanalyze the data reported in this paper is available from the lead contact upon request.

- ChIP-qPCR.

The A549 and H358 cell lines were incubated in low (1 mmol/L) glucose and transfected as previously described [4]. Chromatin was isolated as described previously, starting from 15 × 10^6^ cells. Before immunoprecipitation, an aliquot of chromatin extract was taken as input to be used as a control for qPCR. ChIP was carried out by overnight incubation of chromatin at 4 °C with 50 μL of Dynabeads Protein G (Thermo Fisher Scientific, #10003D), precoated with 5 μg of anti-EZH2 (CST, #5246). The bead washing steps, DNA elution, and extraction were performed as previously described [4]. Before DNA elution, an aliquot of beads for each condition was conserved for Western blot assay, resuspended in sample buffer, and boiled at 90 °C for 5 min. A total of 0.2 ng of DNA was used to amplify the *EGLN3* promoter region. Data analysis was presented as percentages of input. The experiment was repeated a second time, and the data were pooled as a biological replicate.

- meRIP (m6A-RIP).

To enrich the N6-methyladenosine (m6A) modified RNA in immunoprecipitation protocols, the EpiMark N6-Methyladenosine Enrichment Kit (NEB, Ipswich, MA, USA, #E1610S) was used.

A549 and H358 cell lines were cultured in both physiological (5 mmol/L) and low-glucose (1 mmol/L) conditions for 5 days. On day 5, RNA was extracted and treated with DNase I.

Total RNA was fragmented by incubation with RNA fragmentation buffer (100 mM Tris-HCl pH 7.0, 100 mM ZnCl_2_) and heated for 3 min to achieve fragments around 200 nt in length. The reaction was stopped with EDTA, and RNA was purified using phenol/ chloroform/ isoamyl alcohol, followed by ethanol precipitation. RNA fragmentation was confirmed by gel electrophoresis.

Approximately 1 µg of purified RNA was set aside and used as an Input control. For the immunoprecipitation step, about 200 µg of RNA was incubated with N6-Methyladenosine antibody and negative control, both previously bound to the Protein G Magnetic Beads, for 2 h at 4 °C.

After immunoprecipitation, RNA was purified, a cDNA reaction was performed on all samples, and RT-qPCR was run. Data analysis was presented as percentages of input.

The experiment was repeated three times, and the data were pooled as a biological replicate.

- Quantification and statistical analysis.

Data are presented as mean ± standard error of the mean (SEM) unless otherwise indicated. GraphPad Prism 8.0 was used for statistical analysis. For comparisons between two groups, unpaired two-tailed Student’s *t*-tests (parametric or nonparametric as appropriate) were used. *p* < 0.05 was considered statistically significant.

## 3. Results

### 3.1. FTO Is Required to Maintain Cell Differentiation in Lung Cancer Cells

**Figure 1:**
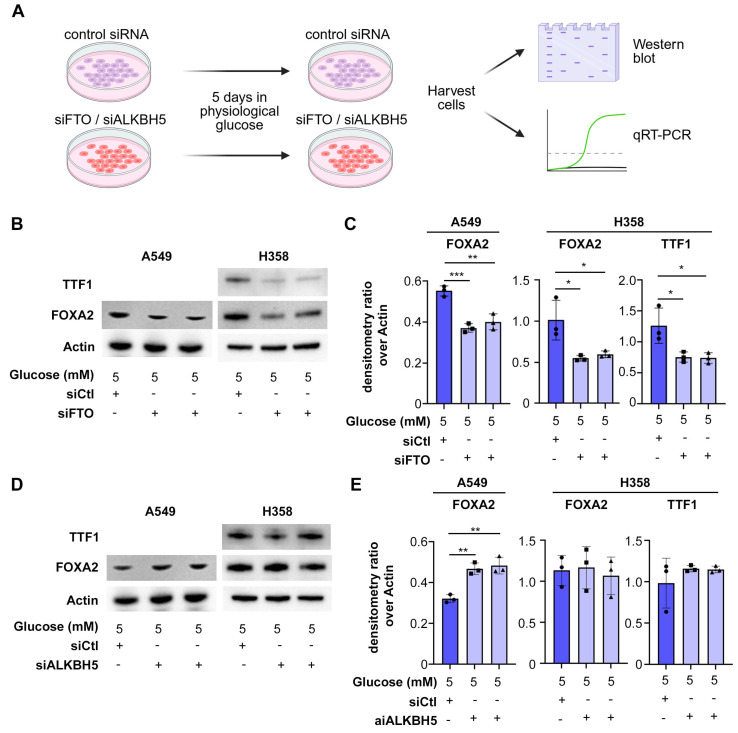
RNA demethylase FTO, but not ALKBH5, is required to maintain protein markers of cell differentiation in LUAD. (**A**) Lung adenocarcinoma cell lines A549 and H358 were incubated in physiological concentration of glucose and transfected with either control siRNA (siCtl) or two different siRNAs targeting *FTO* and *ALKBH5*, followed by Western blot and qRT-PCR (the qPCR results are presented in Figure 2). (**B**) Representative Western blot scans for differentiation markers *TTF1* and *FOXA2* (*TTF1* is not expressed in A549 cells) in *FTO*-knockdown cells. (**C**) Quantification of the Western blot signal (normalized by β-Actin). (**D**) Representative Western blot scans for differentiation markers *TTF1* and *FOXA2* (*TTF1* is not expressed in A549 cells) in *ALKBH5*-knockdown cells. (**E**) Quantification of the Western blot signal (normalized by β-Actin). All experiments were performed in biological triplicate and the data are shown as mean ± SEM; *p* values were determined by Student’s *t*-test; * *p* < 0.05, ** *p* < 0.01, *** *p* < 0.001.

**Figure 2:**
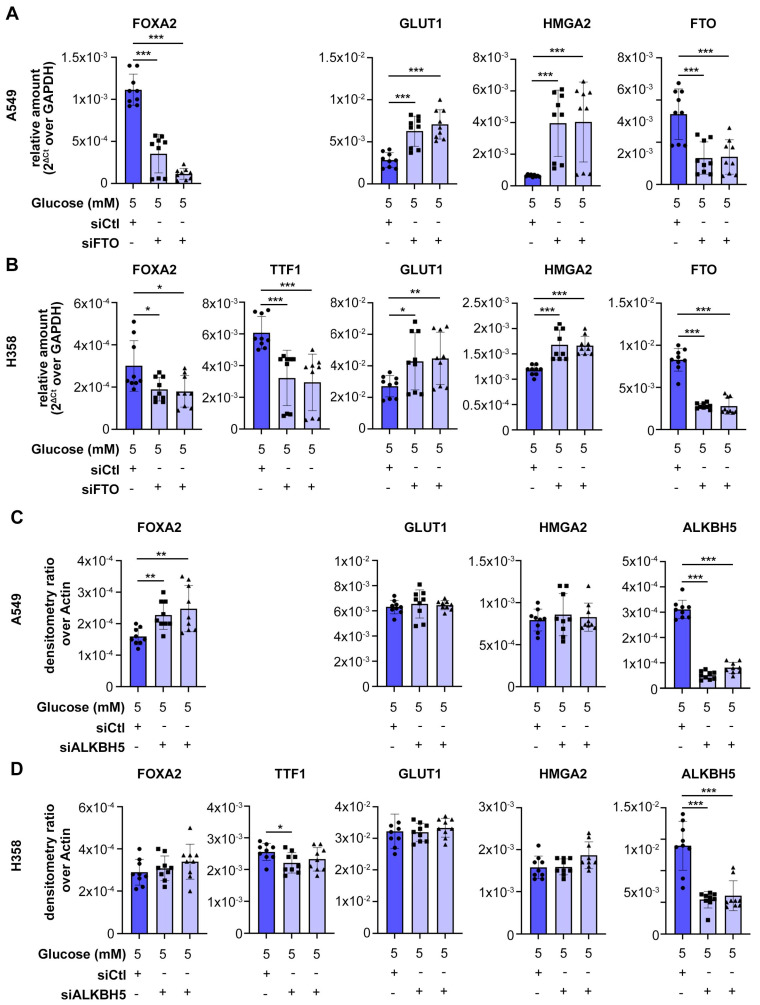
RNA demethylase FTO, but not ALKBH5, is required to maintain RNA markers of cell differentiation in LUAD. Lung adenocarcinoma cell lines A549 and H358 were incubated in physiological concentration of glucose and transfected with either control siRNA (siCtl) or two different siRNAs targeting *FTO* and *ALKBH5* (See experimental scheme in Figure 1A). (**A**,**B**) RT-qPCR in *FTO*-knockdown cells for differentiation markers *FOXA2* and *TTF1*, de-differentiation markers *GLUT1* and *HMGA2*, and *FTO* to confirm the efficiency of knockdown in A549 (**A**) and H358 (**B**) cells. (**C**,**D**) RT-qPCR in *ALKBH5*-knockdown cells for differentiation markers *FOXA2* and *TTF1*, de-differentiation markers *GLUT1* and *HMGA2*, and *ALKBH5* to confirm the efficiency of knockdown in A549 (**C**) and H358 (**D**) cells. All experiments were performed in biological triplicate and the data are shown as mean ± SEM; *p* values were determined by Student’s *t*-test; * *p* < 0.05, ** *p* < 0.01, *** *p* < 0.001.

We previously showed that glucose deprivation causes dedifferentiation of lung adenocarcinoma (LUAD) due to alpha-ketoglutarate (α-KG) deficit. α-KG is co-factor of several enzymes in the cell, including DNA and histone demethylases, as well as RNA demethylases [9,16,17,18,19,20]. To investigate the role of RNA demethylases in the regulation of cell differentiation in lung cancer, we depleted the two known m6A RNA demethylases, *FTO* and *ALKBH5*, in two different human LUAD cell lines: A549 and H358. We measured the effect on differentiation by Western blot and RT-PCR for the following markers of cell differentiation (Figure 1A): *FOXA2* and *TTF-1*, well-known markers of differentiation in LUAD [21,22]; *HMGA2*, a driver of poorly differentiated LUAD [4,22]; GLUT1, a marker of poorly differentiated LUAD [23]. These experiments were performed in physiological concentrations of glucose (5 mM). Western blot analysis showed that knockdown of *FTO*, but not *ALKBH5*, reduced expression of differentiation markers *TTF1* and *FOXA2* in H358 cells, and *FOXA2* in A549 cells (which are less differentiated and lack *TTF1* expression) (Figure 1B–E). RT-qPCR confirmed that knockdown of *FTO*, but not *ALKBH5*, reduced mRNA levels of FOXA2 in A549 cells and both *FOXA2* and *TTF1* in H358 cells (Figure 2). *FTO* knockdown, but not *ALKBH5* knockdown, increased expression of dedifferentiation markers *GLUT1* and *HMGA2* (Figure 2). RT-qPCR confirmed efficient knockdown of *FTO* and of *ALKBH5* with two independent siRNAs (Figure 2). These data show that *FTO*, but not *ALKBH5*, is required to maintain cell differentiation in LUAD cells. We hypothesized that *FTO* inactivation contributes to glucose starvation–induced dedifferentiation, a phenomenon we previously described [4].

### 3.2. Long Non-Coding RNAs Are Upregulated by Glucose Deprivation

**Figure 3:**
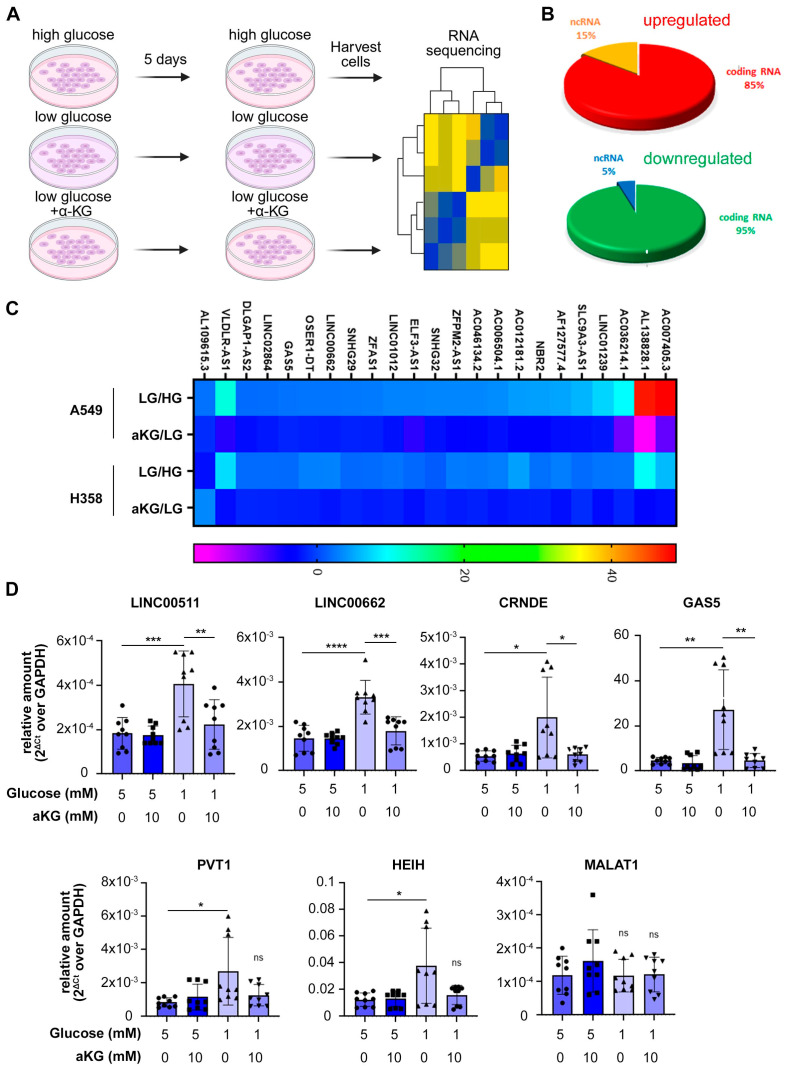
Long non-coding RNAs are upregulated by low glucose. (**A**) Scheme of the experiment. Cells were cultured in either high glucose (HG) or low glucose (LG) for 5 days, with or without supplementation with α-KG (α-KG), followed by RNA extraction for RNA sequencing. (**B**,**C**) RNA sequencing was performed and presented in our previous publication [4]. (**B**) Numbers of long non-coding RNAs up- and downregulated by low glucose in A549 cells. (**C**) Heat map of lncRNAs that are upregulated by low glucose and rescued by α-KG in both A549 and H358 cells. (**D**) A549 cells were incubated in physiological (5 mM) or in low (1 mM) glucose concentrations for 5 days, with or without supplementation of α-KG (10 mM), followed by RT-PCR to confirm that lncRNAs known to interact with FTO and EZH2 are regulated by glucose deprivation and rescued by α-KG. Data in (**D**) are shown as mean ± SEM from at least three biological replicates; *p* values were determined by Student’s *t*-test; * *p* < 0.05, ** *p* < 0.01, *** *p* < 0.001, **** *p* < 0.0001.

To investigate whether RNA methylation contributes to glucose starvation–induced dedifferentiation, we re-analyzed our previously published RNA sequencing data from A549 cells cultured in high (10 mM) or low (1 mM) glucose. A 1 mM glucose concentration mimics the measured intra-tumoral glucose concentration in solid tumors [24,25]. Interestingly, 15% of genes upregulated under low glucose were long non-coding RNAs (lncRNAs), compared with only 5% of downregulated genes (Figure 3A), suggesting that glucose deprivation preferentially stabilizes specific lncRNAs. Twenty-one lncRNAs were concordantly upregulated by glucose deprivation in both A549 and H358 cells, and their induction was abolished by supplementation with 10 mM dimethyl-α-ketoglutarate (dm-αKG) (Figure 3B). The full RNA sequencing dataset was previously published [4]. Table 1 lists the lncRNAs upregulated by glucose deprivation in both cell lines and rescued by dm-αKG.

In our previous publication, we showed that unbalanced activity of histone methyl-transferase EZH2 is a major driver of starvation-induced de-differentiation [4]. EZH2 activity is regulated by lncRNAs, which facilitate its recruitment to target gene promoters [26,27,28]. To investigate the interaction of the glucose starvation-induced lncRNAs with EZH2, we interrogated the LncTarD [29] and StarBase [30] databases of functional lncRNA interactions. We found that six of the twenty-two glucose starvation-induced lncRNAs listed in Table 1 were already known to interact with both EZH2 and FTO (Table 2). RT-PCR confirmed that these EZH2-interacting lncRNAs (*LINC00511*, *LINC00662*, *CRNDE*, *GAS5*, *PVT1*, *HEIH*) were upregulated by low glucose and rescued by dm-α-KG in A549 (Figure 3C) and H358 (Figure S1A) cells, whereas a control lncRNA, *MALAT1*, was not regulated by low glucose nor by dm-α-KG. These data show that EZH2-interacting lncRNAs are upregulated by glucose deprivation. Since FTO is required for maintenance of cell differentiation, we hypothesized that the upregulation of these lncRNAs is due to changes in RNA methylation.

### 3.3. Certain lncRNAs Are Hypermethylated in Low Glucose

**Figure 4:**
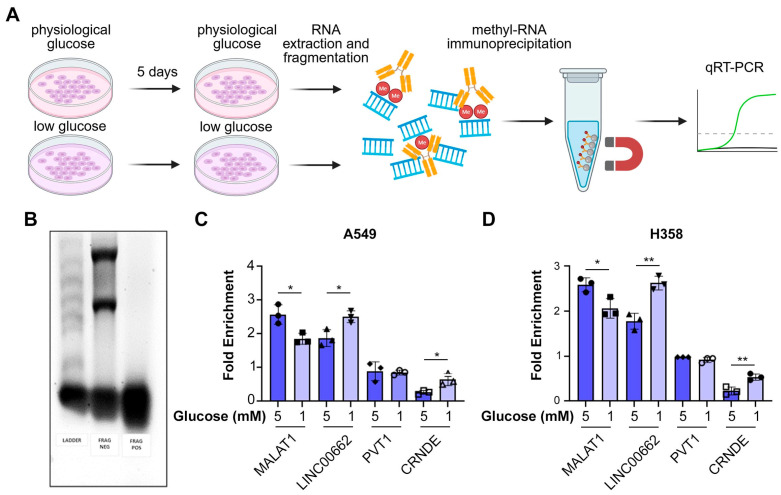
Glucose deprivation induces lncRNA methylation. (**A**) Scheme of the experiment. A549 and H358 cells were incubated in either physiological (5 mM) or low (1 mM) glucose for 5 days, followed by RNA extraction, RNA fragmentation, and methylated RNA immunoprecipitation (MeRIP). (**B**) Gel electrophoresis to confirm RNA fragmentation, (**C**,**D**) RT-PCR for the indicated lncRNAs showing the enrichment of lncRNAs in the immunoprecipitate. Data are presented as mean ± SEM; *p* values were determined by Student’s *t*-test; * *p* < 0.05; ** *p* < 0.01.

To investigate whether glucose deprivation alters N6-methyladenosine (m6A) methylation of the lncRNAs induced by glucose starvation, we performed methylated RNA immunoprecipitation (MeRIP) in A549 and H358 cells cultured in physiological (5 mM) or low (1 mM) glucose (Figure 4A). RNA fragmentation was verified by gel electrophoresis (Figure 4B). Two starvation-induced lncRNAs, *LINC00662* and *CRNDE*, showed significantly higher m6A methylation in low compared with physiological glucose, whereas *PVT1* and the control lncRNA *MALAT1* was less methylated in low glucose (Figure 4C,D). The remaining lncRNAs were below the detection threshold. These findings indicate that glucose deprivation selectively enhances m6A methylation of specific lncRNAs, linking nutrient stress to epitranscriptomic remodeling.

### 3.4. lncRNA LINC00662 Promotes EZH2 Recruitment on Target Gene Promoter in Low Glucose

**Figure 5:**
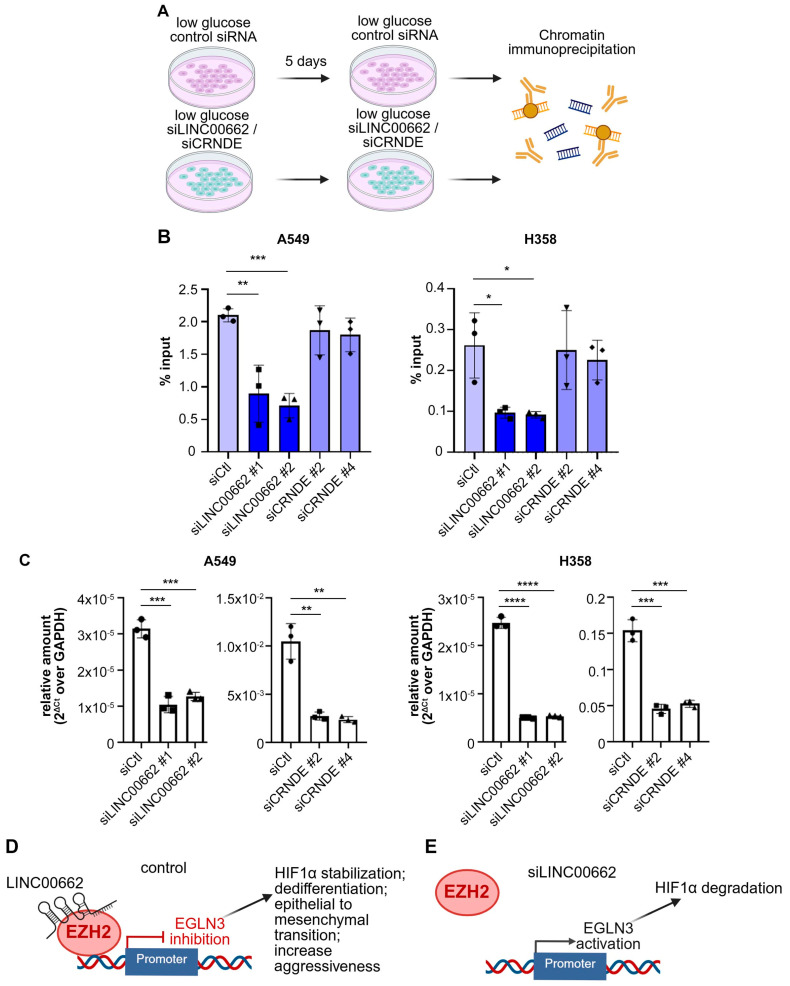
LINC00662 is required for EZH2 recruitment under low glucose. (**A**) Scheme of the experiment. A549 and H358 cells were incubated in low glucose (1mM) for 5 days, with or without transfection of either control or siRNAs targeting *LINC00662* and *CRNDE*. Chromatin immunoprecipitation (ChIP) was performed with an anti-EZH2 antibody, followed by RT-qPCR to detect EZH2 recruitment on target gene *EGLN3* [4]. (**B**) EZH2 recruitment on the *EGLN3* promoter. (**C**) RT-PCR to measure the efficiency of siRNA transfection. Data are presented as mean ± SEM from ≥3 biological replicates; *p* values were determined by Student’s *t*-test; * *p* < 0.05, ** *p* < 0.01, *** *p* < 0.001, **** *p* < 0.0001. (**D**,**E**) Interpretation of the results. (**D**) In the control siRNA sample, LINC00662 promotes EZH2 recruitment on the promoter of target gene *EGLN3*. We have previously shown that *EGLN3* repression by EZH2 in low glucose triggers HIF1α stabilization, dedifferentiation, epithelial-to-mesenchymal transition, and increased aggressiveness [4]. (**E**) When we knock down *LINC00662*, the recruitment of EZH2 on the *EGLN3* promoter in low glucose is hindered.

Since *LINC00662* and *CRNDE* are reported to interact with EZH2 and facilitate its recruitment to target gene promoters, we hypothesized that these lncRNAs may be required for EZH2 recruitment under low-glucose conditions. We previously showed that EZH2 recruitment to the promoter of the prolyl hydroxylase gene *EGLN3* drives starvation-induced dedifferentiation in A549 and H358 cells [4]. We therefore tested the effect of *LINC00662* and *CRNDE* knockdown on EZH2 recruitment to the *EGLN3* promoter. Chromatin immunoprecipitation (Figure 5A) showed that *LINC00662*, but not *CRNDE*, is required for full recruitment of EZH2 on the *EGLN3* promoter in low glucose in both A549 and H358 cells (Figure 5B). The efficiency of lncRNA knockdown was confirmed by RT-PCR (Figure 5C). This result suggests that LINC00662 is required for recruitment of EZH2 on the EGLN3 promoter in response to glucose deprivation (Figure 5D,E). We have previously showed that this event triggers HIF1α stabilization, epithelial-to-mesenchymal transition, and transition to an aggressive phenotype [4].

## 4. Discussion

We previously showed that glucose starvation causes insufficient activity of α-KG-dependent histone demethylases, accompanied by unbalanced activity of histone methyl-transferase EZH2 [4]. Glucose deprivation increases EZH2 recruitment on the *EGLN3* gene promoter and histone hypermethylation, driving stabilization of HIF1α, dedifferentiation, epithelial-to-mesenchymal transition, and development of an aggressive phenotype [4]. Here, we show that glucose deprivation also causes insufficient activity of RNA m6A demethylases, leading to hypermethylation of certain lncRNAs. One of these lncRNAs, *LINC00662*, is required for recruitment of EZH2 on the *EGLN3* promoter in low glucose. In physiological glucose, a normal activity of m6A demethylases maintains the balance between methylation and demethylation required for maintenance of cell differentiation (Graphical abstract, A). Following glucose deprivation, insufficient activity of α-KG-dependent RNA demethylase FTO causes hypermethylation of lncRNAs, including *LINC00662*, accompanied by upregulation of *LINC00662* and increased recruitment of EZH2 on the *EGLN3* promoter (graphical abstract, B). Given the previously demonstrated role of *EGLN3* repression in driving dedifferentiation and transition to an aggressive phenotype [4], we assume that *LINC00662* stabilization contributes to glucose starvation-induced dedifferentiation.

Our findings underscore the critical role of FTO in maintaining differentiation in LUAD cells. Knockdown of *FTO*, but not *ALKBH5*, reduced differentiation markers (*TTF1*, *FOXA2*) and increased dedifferentiation markers (*GLUT1*, *HMGA2*). This suggests that FTO is specifically required for the maintenance of differentiation in LUAD cells. Although FTO and ALKBH5 belong to the same class of α-KG-dependent dioxygenases, they show significant structural and substrate specificity differences that can explain their different role in regulating cell differentiation in LUAD [43]. *FTO* is downregulated in LUAD relative to adjacent normal tissue, and its reduced expression correlates with poor prognosis [44,45]. Mechanistically, Wnt signaling has been linked to *FTO* repression through EZH2 recruitment to the *FTO* promoter [45]. Therefore, FTO insufficiency can be induced in starvation-induced differentiation via two distinct mechanisms: (1) direct inhibition due to insufficient intracellular concentration of α-KG [4]; (2) transcriptional repression by EZH2 [45], which is overactive under nutrient stress [4]. The specificity of FTO, as opposed to ALKBH5, suggests distinct and non-redundant functions for these RNA demethylases in LUAD.

Upregulation of lncRNAs under glucose deprivation provides further mechanistic insight. A significant proportion of glucose-induced genes were lncRNAs, many of which regulate EZH2 [36], a histone methyltransferase known to drive dedifferentiation in cancer cells [4,5]. We found that several lncRNAs, including *LINC00511*, *LINC00662*, and *CRNDE*, were hypermethylated and upregulated by glucose deprivation in both A549 and H358 cells, and this induction was rescued by supplementation with dimethyl-α-ketoglutarate (dm-α-KG). These results underscore the role of α-KG in regulating RNA methylation. Although we do not know whether hypermethylation is responsible for upregulation of these lncRNAs, m6A methylation has been described as a powerful regulator of RNA stability and function [46,47]. Future experiments will be required to test the hypothesis that deficient FTO-mediated demethylation of these lncRNAs determines their stabilization in low glucose. Among the reported lncRNAs, *LINC00662* uniquely facilitated EZH2 recruitment to gene promoters, including *EGLN3*, a known EZH2 target in starvation-induced dedifferentiation [4]. Lv et al. reported that *LINC00662* is enriched in tumor tissue and plasma exosomes of non-small cell lung cancer patients, promoting proliferation, invasion, and migration [48]. Gong et al. reported that *LINC00662* is upregulated in non-small cell lung cancer compared with adjacent normal tissue and promotes a stem cell-like phenotype in lung cancer cells [49]. Yuan et al. showed that over-expression of *LINC00662* in non-small cell lung cancer is driven by copy number amplification and drives tumor growth in vitro and in vivo via recruitment of EZH2 on target gene promoters [40]. These reports confirm the clinical relevance of *LINC00662* as a pathogenic driver and a potential prognostic factor in human non-small cell lung cancer [40].

Our methylation analysis revealed that specific starvation-induced lncRNAs, including *LINC00662* and *CRNDE*, undergo significant hypermethylation under glucose deprivation. This suggests that RNA methylation, normally regulated by FTO, plays a crucial role in stabilizing these lncRNAs during nutrient stress. Hypermethylation may increase lncRNA stability and strengthen their interaction with EZH2, thereby promoting chromatin remodeling and gene expression changes that favor a dedifferentiated phenotype. In contrast, the absence of methylation changes in *PVT1* and the reduced methylation of control lncRNA *MALAT1* supports the specificity of this mechanism. Together, these findings define a mechanistic link between nutrient stress, epitranscriptomic remodeling, and chromatin regulation in LUAD, positioning *LINC00662* as a key mediator of EZH2-driven dedifferentiation. This mechanism is important as EZH2 inhibition therapy has been suggested to promote the response to immunotherapy in pre-clinical models [50] and is being investigated in several clinical trials for non-small cell lung cancer (NCT05467748, NCT06644768, NCT06022757, NCT05023655).

## 5. Conclusions

Collectively, these findings provide new insights into the molecular network governing LUAD cell differentiation and dedifferentiation in response to glucose deprivation. The identification of FTO as a key regulator of RNA methylation, together with the upregulation of specific lncRNAs that interact with EZH2, highlights new avenues for therapeutic intervention. Targeting FTO or the RNA methylation machinery may restore differentiation and counteract dedifferentiation in LUAD cells, offering a novel therapeutic strategy. Moreover, the discovery that lncRNAs play an active role in mediating these processes emphasizes the importance of non-coding RNAs in cancer biology, offering a new dimension to the development of therapeutic approaches. Further studies are required to establish the clinical relevance of these findings and to validate the potential of targeting the FTO–lncRNA–EZH2 axis in LUAD treatment. By linking metabolic stress to epitranscriptomic regulation and EZH2-mediated chromatin control, this study reveals novel vulnerabilities in LUAD that may be exploited for precision therapies. EZH2 is an increasingly recognized therapeutic target for lung cancer. The elucidation of the dedifferentiation mechanism that links nutrient deprivation with lncRNA methylation/expression and recruitment of EZH2 on the promoter of target genes is likely to offer new targets to predict the response to EZH2 inhibitors or to provide a rationale for combination treatments that can improve the efficacy of EZH2 inhibition. For instance, reactivation of FTO by therapeutic strategies that increase intracellular α-KG could prevent EZH2 activation and the triggering of starvation-induced dedifferentiation.

## Figures and Tables

**Table 1:** LncRNAs upregulated by glucose starvation and rescued by α-KG supplementation in A549 and H358 cells.

GENE ID	A549				H358			
	LG/HG		α-KG/LG		LG/HG		α-KG/LG	
	FC	padj	FC	padj	FC	padj	FC	padj
AC007405.3	48.8	2.96 × 10^−3^	−8.0	5.00 × 10^−2^	6.0	6.93 × 10^−4^	−2.6	1.17 × 10^−1^
AL138828.1	47.2	1.20 × 10^−5^	−16.7	1.02 × 10^−4^	9.9	2.75 × 10^−3^	−3.0	2.09 × 10^−1^
AC036214.1	9.5	1.79 × 10^−2^	−8.4	2.63 × 10^−2^	2.4	2.85 × 10^−3^	−1.6	2.02 × 10^−1^
LINC01239	7.2	2.32 × 10^−3^	−2.2	1.48 × 10^−1^	2.3	2.65 × 10^−2^	−2.6	1.28 × 10^−2^
SLC9A3-AS1	5.6	6.21 × 10^−10^	−2.4	1.88 × 10^−3^	1.9	4.35 × 10^−2^	−1.2	7.94 × 10^−1^
AF127577.4	4.8	4.15 × 10^−6^	−2.6	6.43 × 10^−3^	2.7	6.39 × 10^−3^	−2.5	2.04 × 10^−2^
NBR2	4.5	1.20 × 10^−16^	−3.5	3.82 × 10^−12^	2.0	3.47 × 10^−4^	−1.8	4.75 × 10^−3^
AC012181.2	4.4	1.27 × 10^−2^	−3.8	2.68 × 10^−2^	4.5	1.84 × 10^−2^	−1.9	4.52 × 10^−1^
AC006504.1	3.6	5.58 × 10^−4^	−2.5	1.96 × 10^−2^	2.7	1.77 × 10^−2^	−2.3	8.47 × 10^−2^
AC046134.2	3.3	2.34 × 10^−4^	−3.0	1.06 × 10^−3^	2.4	3.18 × 10^−2^	−1.7	2.67 × 10^−1^
ZFPM2-AS1	3.2	5.31 × 10^−5^	−3.3	6.44 × 10^−5^	2.6	1.27 × 10^−3^	−2.1	3.26 × 10^−2^
SNHG32	3.2	2.41 × 10^−12^	−2.4	2.18 × 10^−7^	1.1	7.51 × 10^−1^	−1.6	3.91 × 10^−2^
ELF3-AS1	3.0	1.10 × 10^−3^	−5.8	1.64 × 10^−7^	1.7	2.03 × 10^−1^	−2.5	2.75 × 10^−2^
LINC01012	3.0	1.29 × 10^−4^	−2.4	2.69 × 10^−3^	2.2	1.03 × 10^−2^	−1.8	1.00 × 10^−1^
ZFAS1	2.6	1.39 × 10^−12^	−1.7	1.15 × 10^−4^	1.9	2.41 × 10^−5^	−2.0	7.39 × 10^−6^
SNHG29	2.5	7.66 × 10^−9^	−1.6	4.10 × 10^−3^	1.6	7.49 × 10^−3^	−1.6	1.14 × 10^−2^
LINC00662	2.3	2.97 × 10^−4^	−2.0	5.01 × 10^−3^	3.0	9.27 × 10^−6^	−2.2	2.82 × 10^−3^
OSER1-DT	2.3	2.49 × 10^−4^	−1.8	9.82 × 10^−3^	2.9	2.91 × 10^−5^	−1.9	2.43 × 10^−2^
GAS5	2.2	7.72 × 10^−7^	−1.5	1.46 × 10^−2^	1.8	1.27 × 10^−3^	−1.8	2.32 × 10^−3^
LINC02864	1.9	4.51 × 10^−5^	−2.2	1.97 × 10^−6^	1.9	8.05 × 10^−4^	−1.6	2.02 × 10^−2^
DLGAP1-AS2	1.9	1.74 × 10^−2^	−2.5	3.21 × 10^−4^	1.8	3.58 × 10^−2^	−1.5	2.76 × 10^−1^
VLDLR-AS1	11.1	1.15 × 10^−7^	−5.5	1.58 × 10^−4^	7.7	2.60 × 10^−5^	−2.6	8.65 × 10^−2^

A549 and NCI-H358 cell lines were incubated for 5 days in high glucose (HG, 20 mM), low glucose (LG, 1 mM), and low glucose + dimethyl-α-KG (α-KG, 10 mM). Total RNA was extracted and RNA sequencing was performed as previously reported [4]. The full dataset has already been published [4]. Here, we report only the lncRNAs that were significantly upregulated by glucose deprivation and rescued by α-KG consistently in both cell lines. Differential expression was reported as |fold-change| (FC) ≥ 1.5 along with associated adjusted *p* value (padj) ≤ 0.05 computed according to Benjamini–Hochberg. LncRNAs whose FC was significant in at least of the three reported conditions and changed in the same direction in the fourth condition were included as robustly regulated by glucose deprivation in an α-KG-dependent way. Significant fold changes in upregulated genes are in red, significant fold changes in downregulated genes are in green, and non-significant fold changes are in black.

**Table 2:** LncRNAs upregulated by low glucose for which there is evidence in the literature of interaction with EZH2/FTO.

LncRNA	EZH2 Interaction (LncTarD)	FTO Interaction (StarBase)	References
*LINC00511*	YES	YES	LINC00511 interacts with EZH2 [31,32,33]
*GAS5*	YES	YES	GAS5 interacts with EZH2 [34,35,36]. GAS5 is de-methylated by FTO [37,38]
*CRNDE*	YES	YES	CRNDE interacts with EZH2 [39]
*LINC00662*	NO	YES	LINC00662 interacts with EZH2 [40]
*HEIH*	YES	NO	
*PVT1*	YES	YES	PVT1 interacts with EZH2 [41,42]
*MALAT1*	NO	NO	-

The interaction of the reported lncRNAs with EZH2 was searched in LncTarD and StarBase databases. If the interaction was reported in the database, the interaction is reported as “YES”. If the interaction was not reported in the respective database, it is marked as “NO”. In addition to the database search, the interaction between the reported lncRNAs and EZH2 was searched in the literature and reported in the References column. Even if the interaction is not reported in one of the two databases, it can be still validated independently in the literature. *MALAT1* is included as a negative control with no reported interaction with EZH2.

## Data Availability

The RNA-seq raw data are publicly available in ArrayExpress (RRID: SCR_002964) repository under accession number E-MTAB-11253. The original contributions presented in this study are included in the article/Supplementary Materials. Further inquiries can be directed to the corresponding author.

## Supplementary Materials

The following supporting information can be downloaded at https://www.mdpi.com/article/10.3390/biom15111493/s1. Figure S1: LncRNAs are upregulated by low glucose and rescued by dimethyl-alpha-ketoglutarate in H358 cells. Table S1: Commercially available siRNAs used in the project. Table S2: Antibodies used in the project. Table S3: Primer sequences used in the project. Original Western Blots.

## Abbreviations

The following abbreviations are used in this manuscript:

LUAD	Lung adenocarcinoma
α-KG	alpha-ketoglutarate
TCA	Tricarboxylic Acid Cycle
SDS-PAGE	Sodium dodecyl-sulfate Polyacrylamide Gel Electrophoresis
ChIP	Chromatin immunoprecipitation
MeRIP	Methyl-RNA immunoprecipitation
lncRNA	Long non-coding RNA
