# Supplementary material for: Long Non-Coding RNAs Contribute to Glucose Starvation-Induced Dedifferentiation in Lung Adenocarcinoma
Source: Biomolecules. 2025 Oct 23;15(11):1493. doi: 10.3390/biom15111493 (PMC12650432; doi:10.3390/biom15111493)
Supplement: Supplementary file 1 [file biomolecules-15-01493-s001.zip › TableS1-S3.pdf]

Table S1. Commercially available siRNAs used in the project		
**siRNA**	**Company**	**GeneGlobe ID #**
FTO_7 FlexiTube siRNA (1 nmol)	Qiagen	SI04293625
FTO_8 FlexiTube siRNA (1nmol)	Qiagen	SI04337039
ALKBH5_1 FlexiTube siRNA (1nmol)	Qiagen	SI04138869
ALKBH5_2 FlexiTube siRNA (1nmol)	Qiagen	SI04240705
LOC148189_1 FlexiTube siRNA (1 nmol)	Qiagen	SI05138812
LOC148189_2 FlexiTube siRNA (1 nmol)	Qiagen	SI05138819
LOC643911_2 FlexiTube siRNA (1 nmol)	Qiagen	SI02788611
LOC643911_4 FlexiTube siRNA (1 nmol)	Qiagen	SI02788597

Table S2. Antibodies used in the project

Antibodies for Western Blotting	Source	Catalog Number
Rabbit mAb anti-EZH2 (1:1000)	Cell Signaling Technology	Cat#5246
Rabbit mAb anti-FoxA2/HNF3β (1:2000)	Cell Signaling Technology	Cat#8186
Rabbit mAb anti-Thyroid Transcription Factor 1 (TTF-1) (1:2000)	Cell Signaling Technology	Cat#12373
**Antibodies for Chromatin Immunoprecipitation**	**Source**	**Catalog Number**
Rabbit mAb anti-EZH2	Cell Signaling Technology	Cat#5246

Table S3. Primer sequences used in the project

RT-PCR primers

**Gene Name**	**Sequence**
FOXA2	Forward: GGAGCAGCTACTATGCAGAGC<br>Reverse: CGTGTTCATGCCGTTCATCC
TTF1	Forward: ATGTACCGGGACGACTTGGAA<br>Reverse: CAATGCCTGTCAGGGCTAGAA
HMGA2	Forward: CATTGGAGAAAAACGGCCAAG<br>Reverse: TTGCGAGGATGTCTCTTCAGT
GLUT1	Forward: GCTTCCAGTATGTGGAGCAAC<br>Reverse: AGGTCCGGCCTTTAGTCTCA
FTO	Forward: GCT GCT TAT TTC GGG ACC TG<br>Reverse: AGC CTG GAT TAC CAA TGA GGA
ALKBH5	Forward: CGG CGA AGG CTA CAC TTA CG<br>Reverse: CCA CCA GCT TTT GGA TCA CCA
LINC00511	Forward: CGC AAG GAC CCT CTG TTA GG<br>Reverse: GAA GGC GGA TCG TCT CTC AG
LINC00662	Forward: CTA GAA TTC GTA GTC CGG CCG CCC TGT GA<br>Reverse: CTA GGA TCC TCA CCA CGA CCA AGT GCA ATT TAT T
HEIH	Forward: CCT CTT GTG CCC CTT TCT T<br>Reverse: ATG GCT TCT CGC ATC CTA T
MALAT1	Forward: GCT CTG TGG TGT GGG ATT GA<br>Reverse: GTG GCA AAA TGG CGG ACT TT
PVT1	Forward: TGA GAA CTG TCC TTA CGT GAC C<br>Reverse: AGA GCA CCA AGA CTG GCT CT
CRNDE	Forward: TGG ATG CTG TCA GCT AAG TTC AC<br>Reverse: TTC CAG TGG CAT CCT CCT TAT C

ChIP primers

**Gene Name**	**Sequence**
hEGLN3	Forward: CACGATCACCTTCTGACAGGG<br>Reverse: GGGTAAGGGAAAGAAAGATCCTGTA
