# Supplementary figures and images for: Long Non-Coding RNAs Contribute to Glucose Starvation-Induced Dedifferentiation in Lung Adenocarcinoma
Source: Biomolecules. 2025 Oct 23;15(11):1493. doi: 10.3390/biom15111493 (PMC12650432; doi:10.3390/biom15111493)

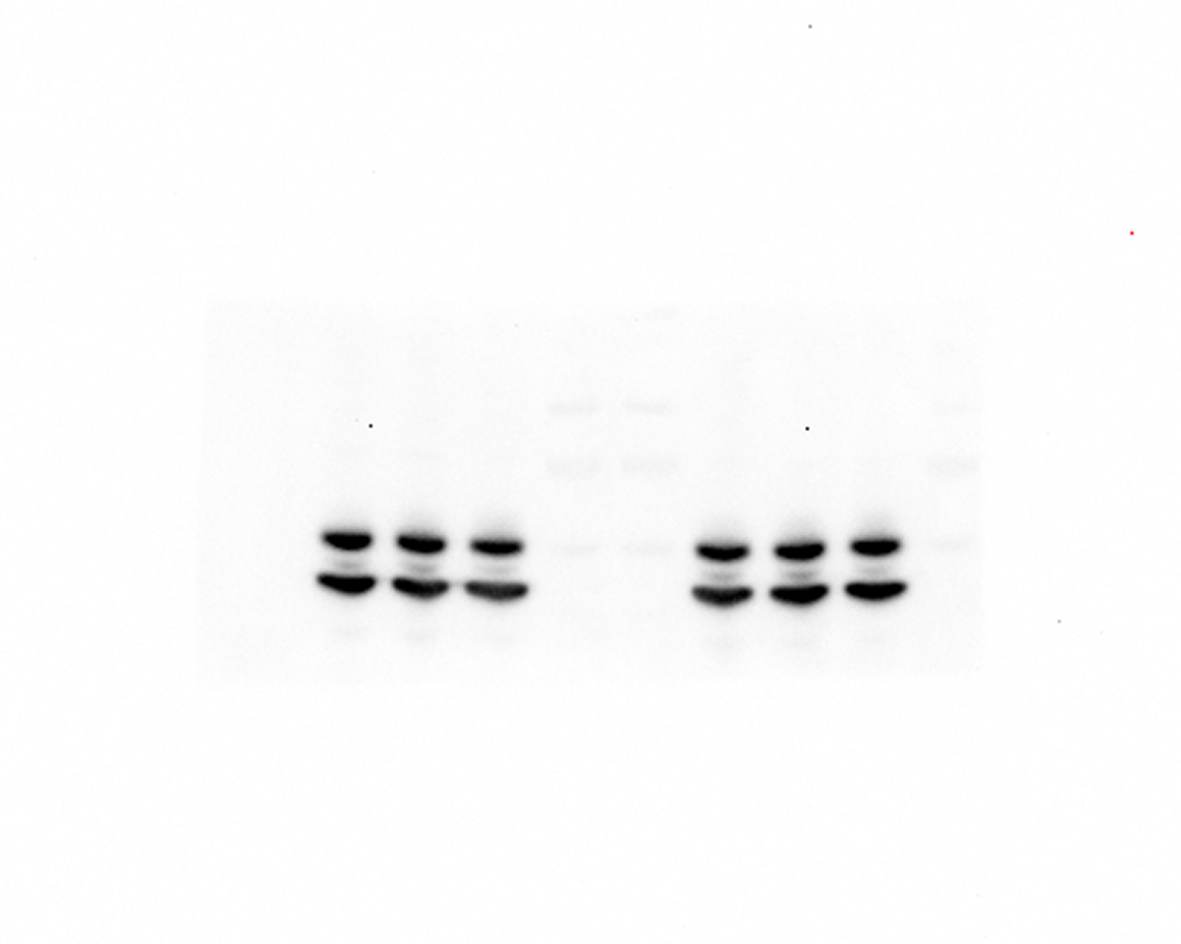

Supplement: Supplementary file 1 [file biomolecules-15-01493-s001.zip › A549_Experiment1_Actin.tif]

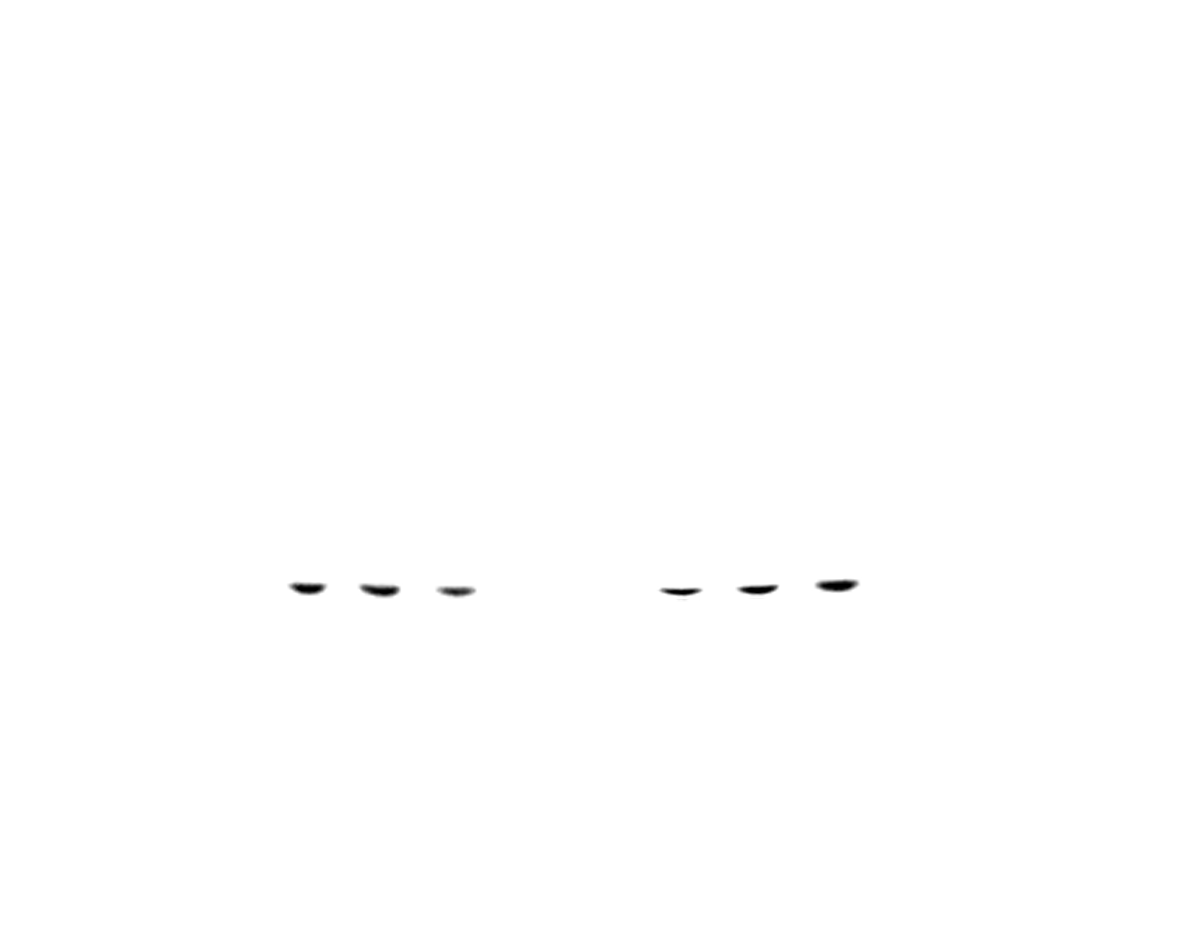

Supplement: Supplementary file 1 [file biomolecules-15-01493-s001.zip › A549_Experiment1_FOXA2.tif]

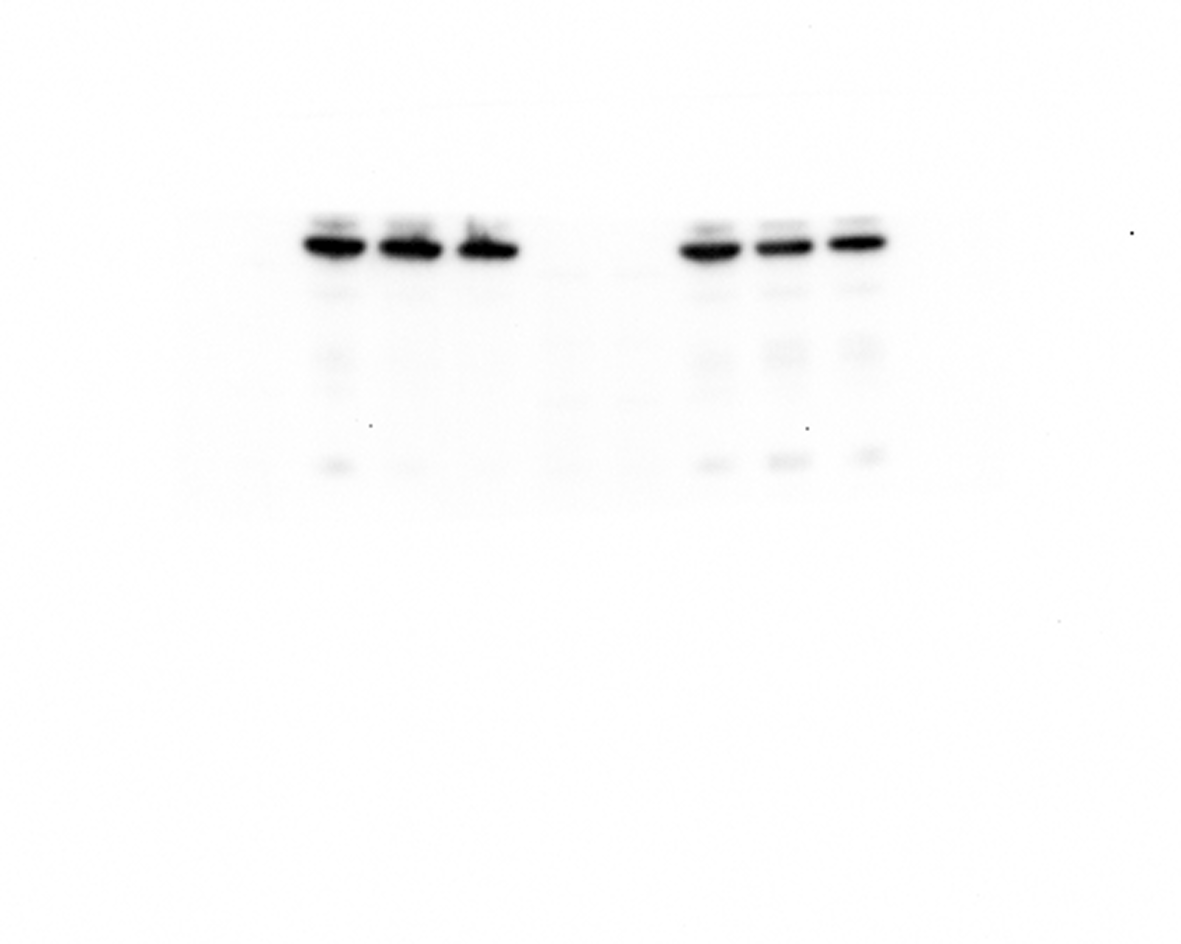

Supplement: Supplementary file 1 [file biomolecules-15-01493-s001.zip › A549_Experiment2_Actin.tif]

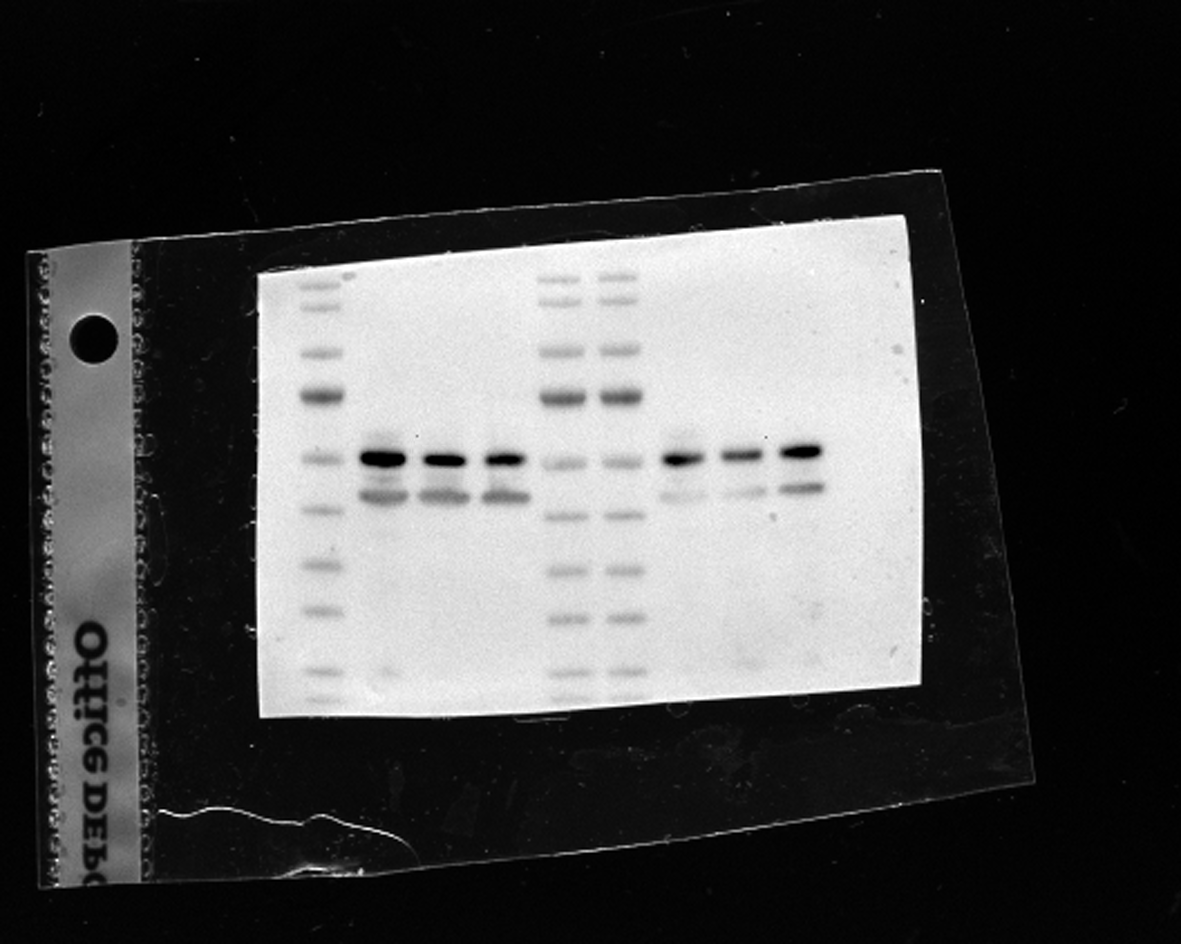

Supplement: Supplementary file 1 [file biomolecules-15-01493-s001.zip › A549_Experiment2_FOXA2.tif]

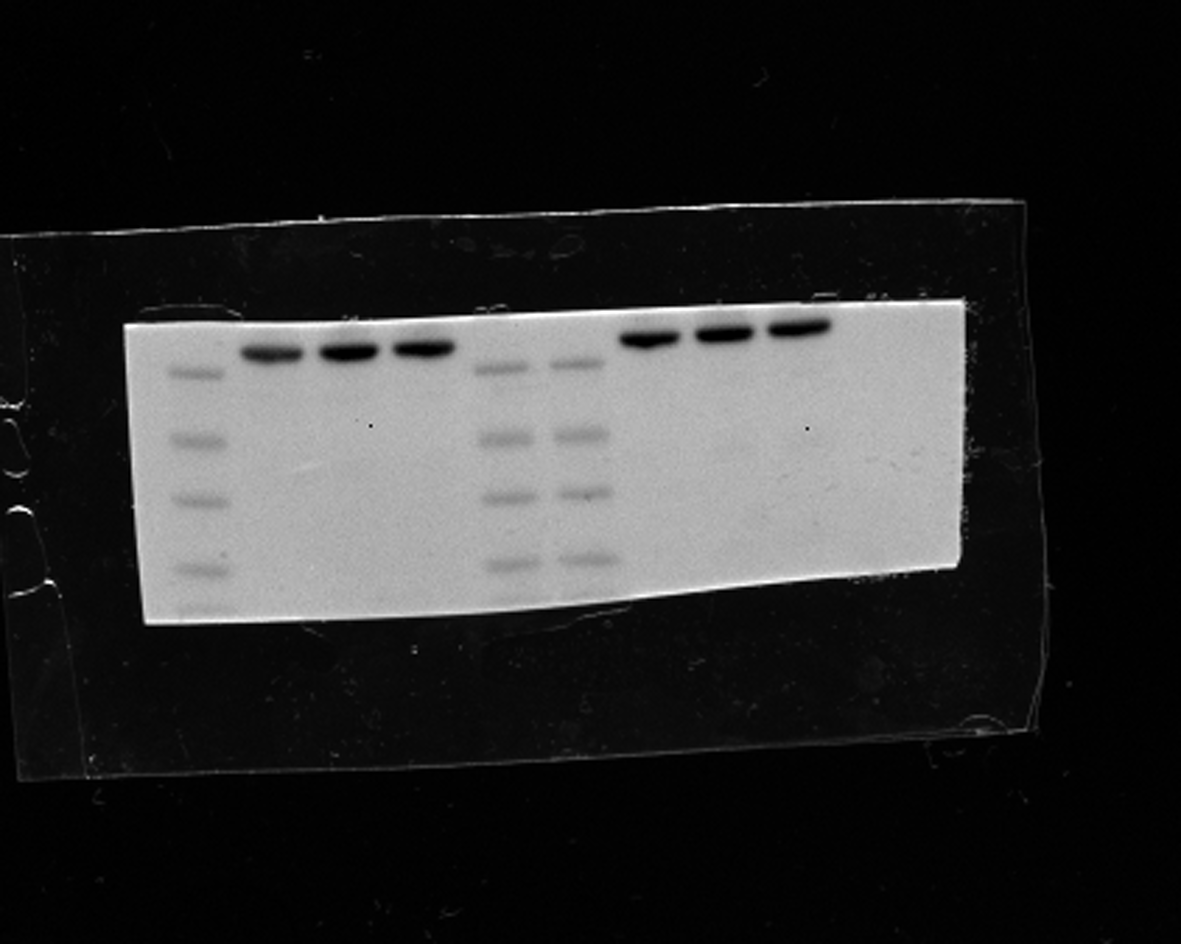

Supplement: Supplementary file 1 [file biomolecules-15-01493-s001.zip › A549_Experiment3_Actin.tif]

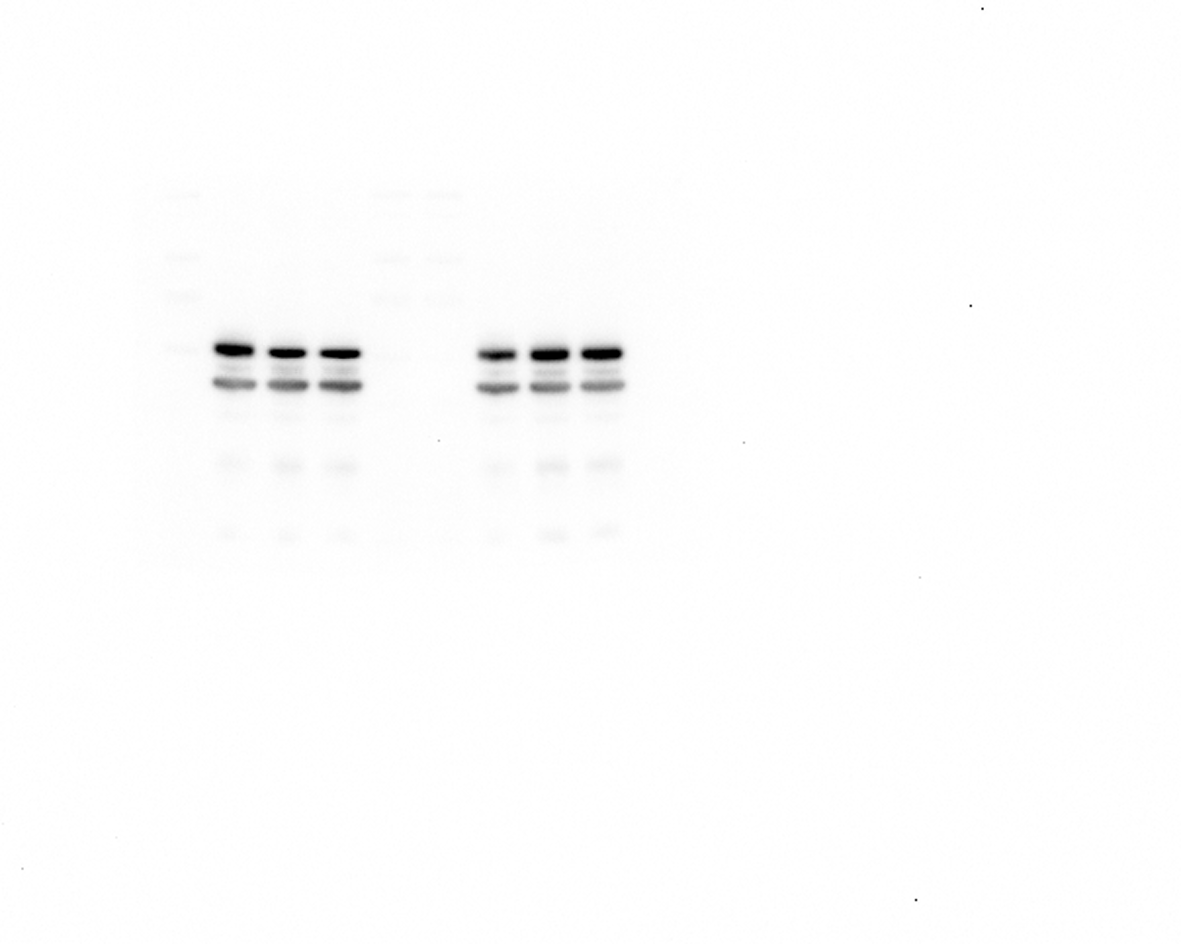

Supplement: Supplementary file 1 [file biomolecules-15-01493-s001.zip › A549_Experiment3_FOXA2.tif]

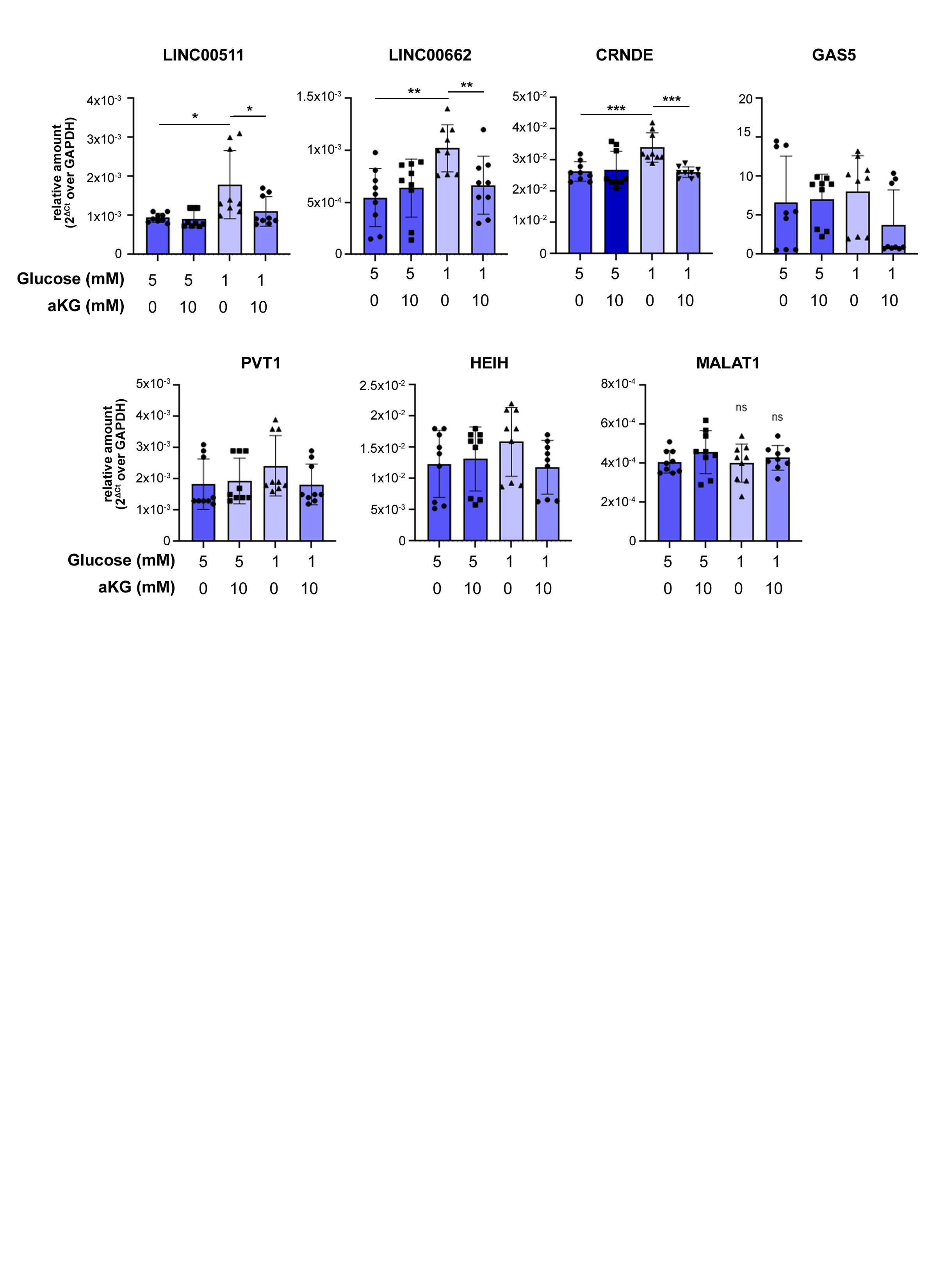

Supplement: Supplementary file 1 [file biomolecules-15-01493-s001.zip › Fig.S1 copy.tif]

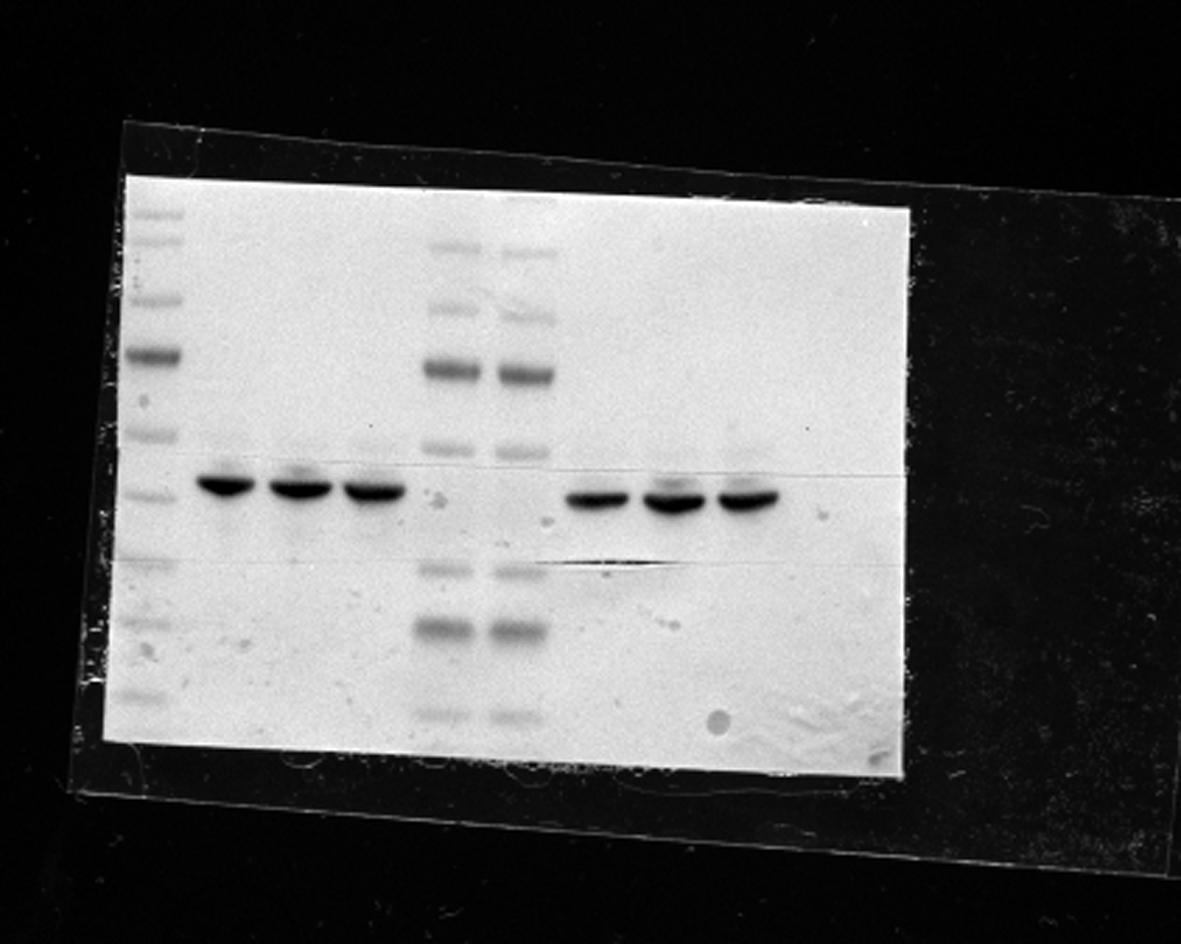

Supplement: Supplementary file 1 [file biomolecules-15-01493-s001.zip › H358_Experiment1_Actin.tif]

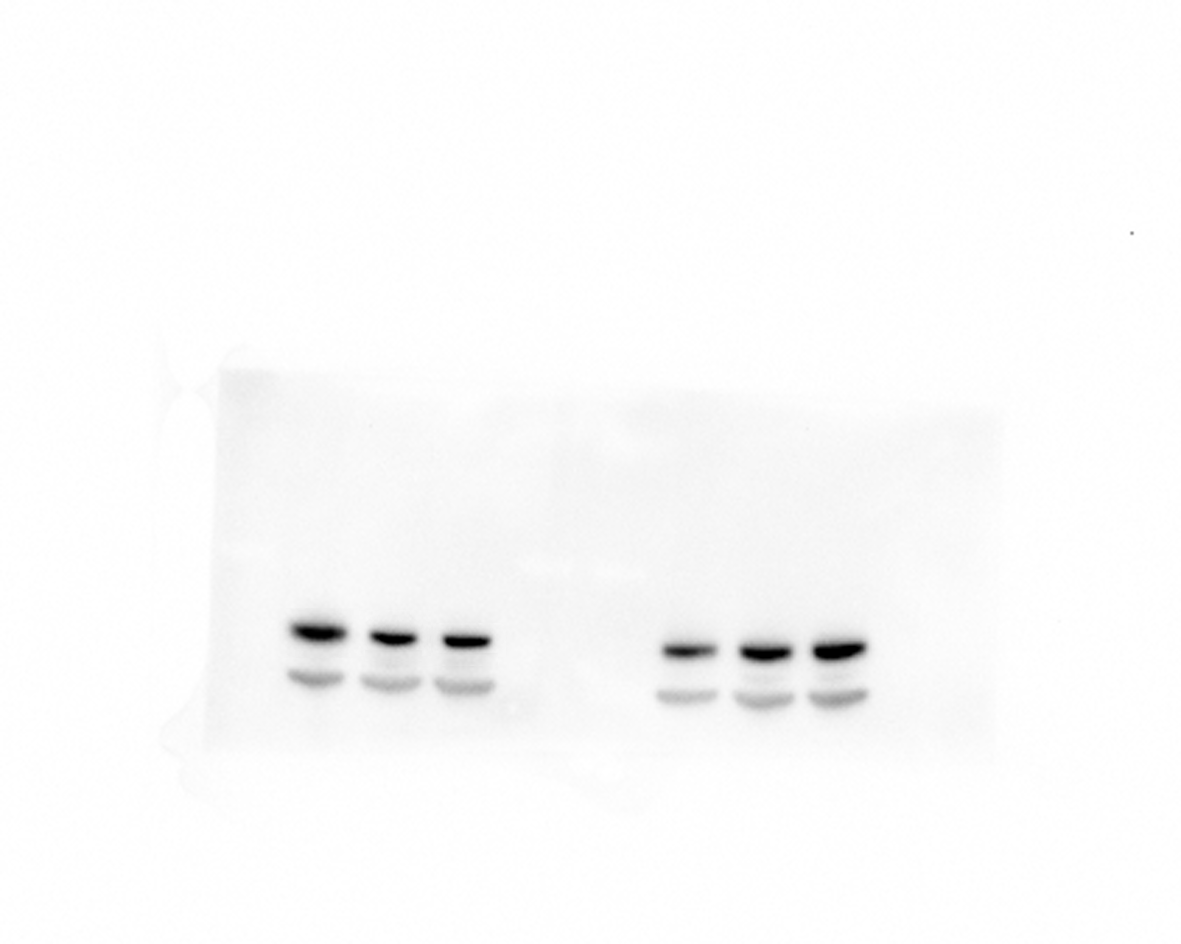

Supplement: Supplementary file 1 [file biomolecules-15-01493-s001.zip › H358_Experiment1_FOXA2.tif]

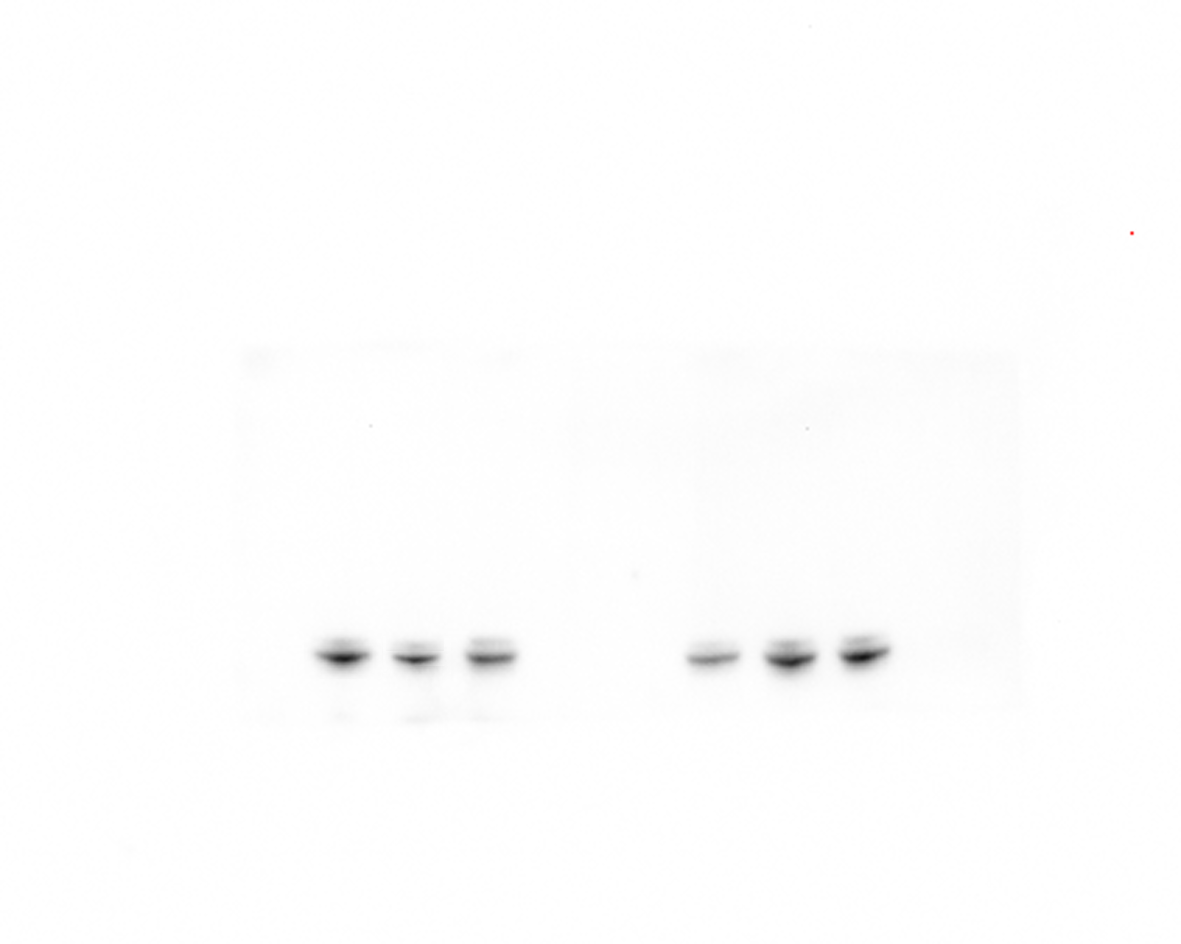

Supplement: Supplementary file 1 [file biomolecules-15-01493-s001.zip › H358_Experiment1_TTF1.tif]

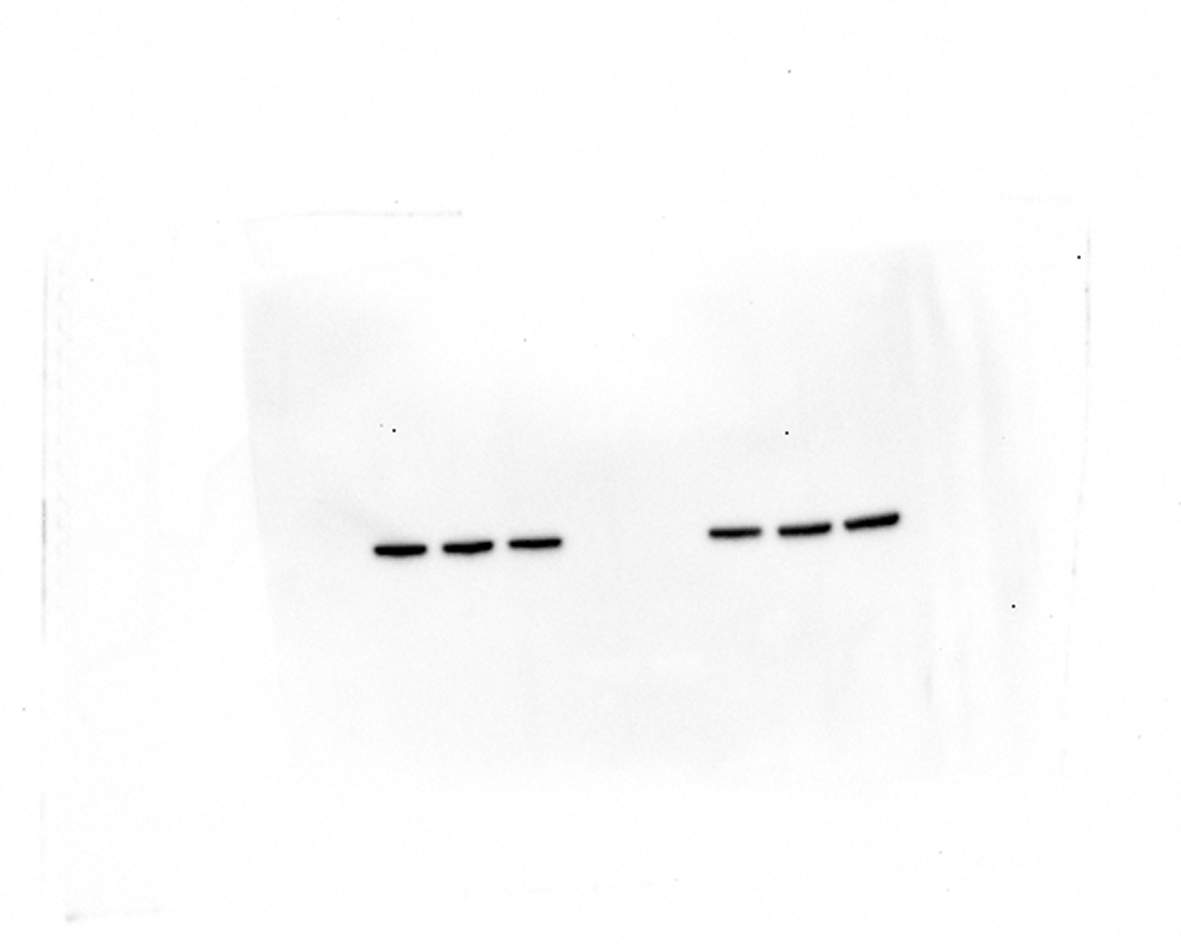

Supplement: Supplementary file 1 [file biomolecules-15-01493-s001.zip › H358_Experiment2_Actin.tif]

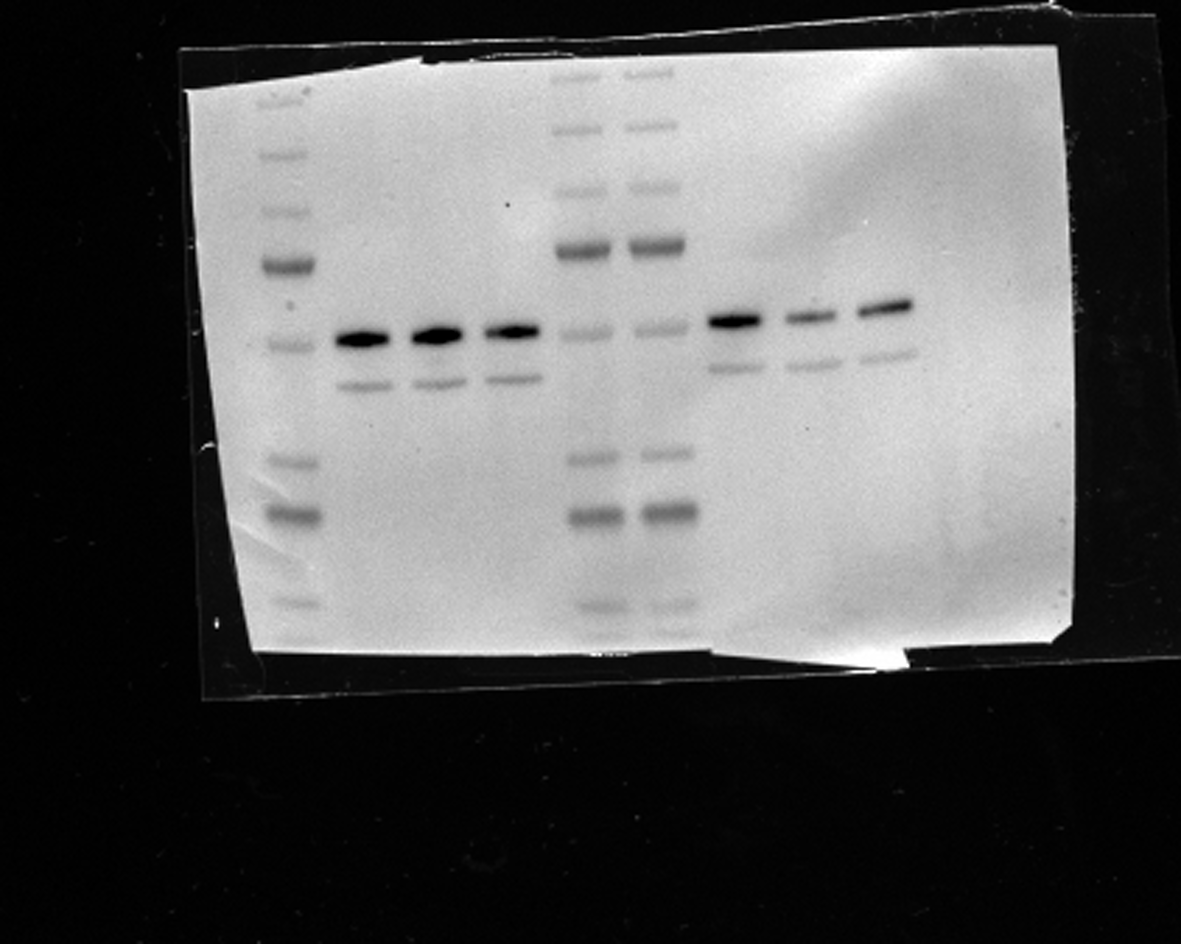

Supplement: Supplementary file 1 [file biomolecules-15-01493-s001.zip › H358_Experiment2_FOXA2.tif]

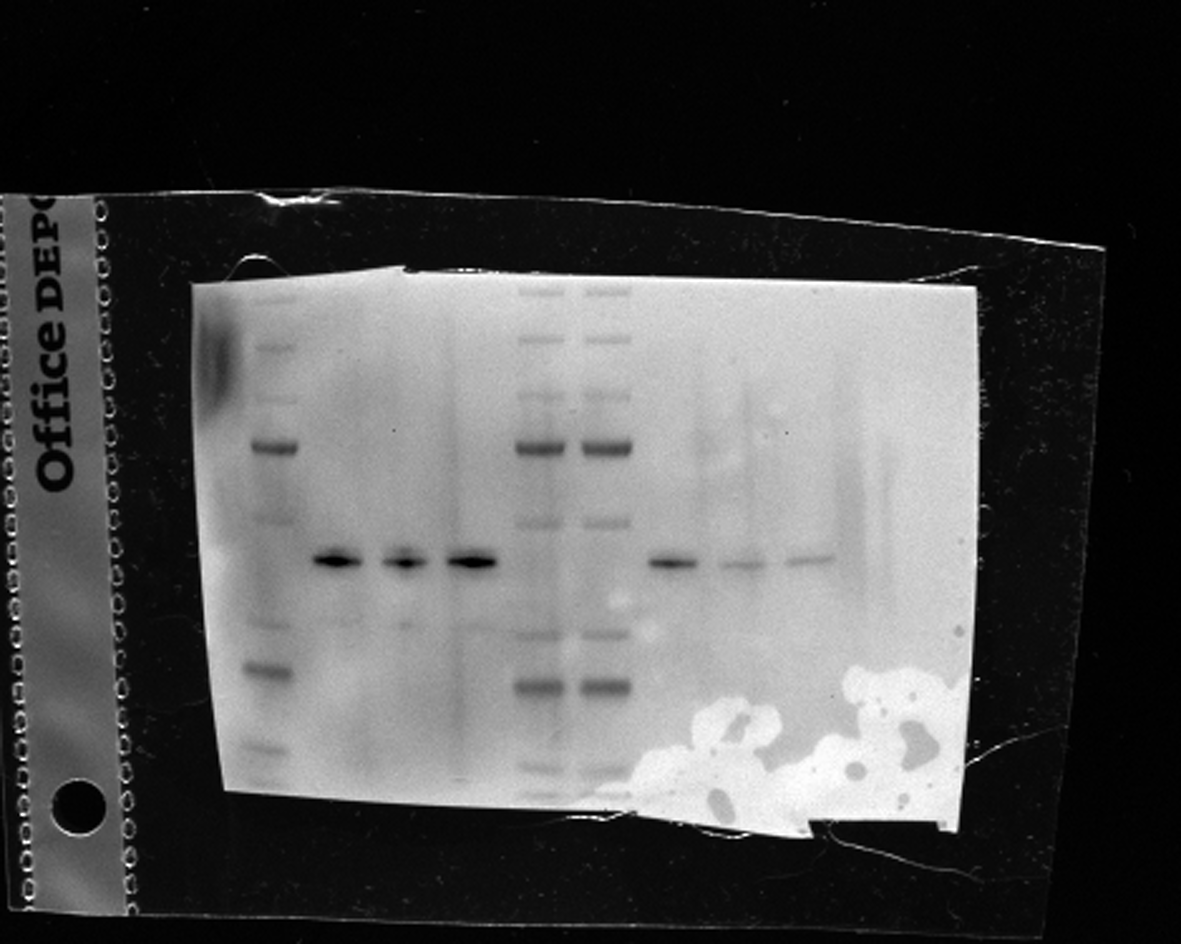

Supplement: Supplementary file 1 [file biomolecules-15-01493-s001.zip › H358_Experiment2_TTF1.tif]

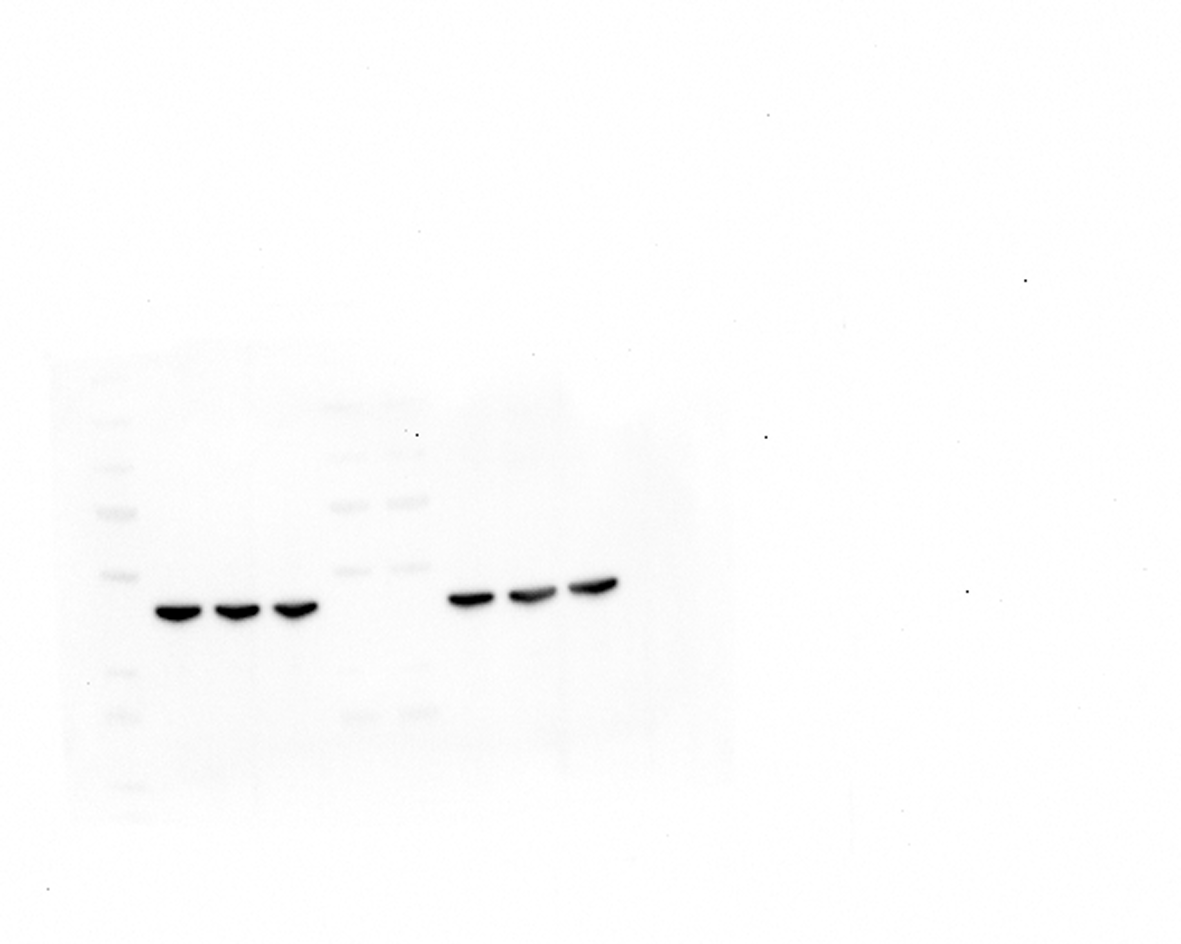

Supplement: Supplementary file 1 [file biomolecules-15-01493-s001.zip › H358_Experiment3_Actin.tif]

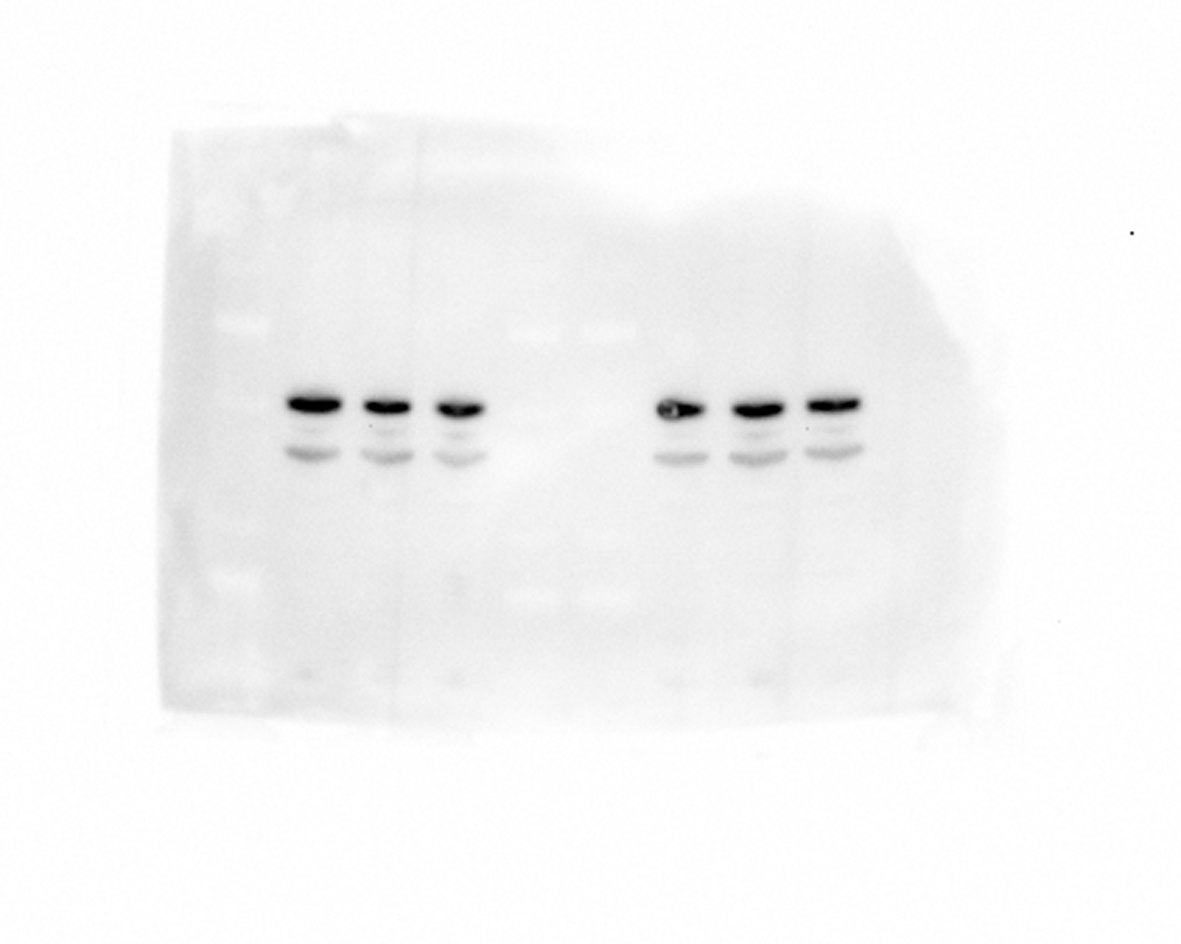

Supplement: Supplementary file 1 [file biomolecules-15-01493-s001.zip › H358_Experiment3_FOXA2.tif]

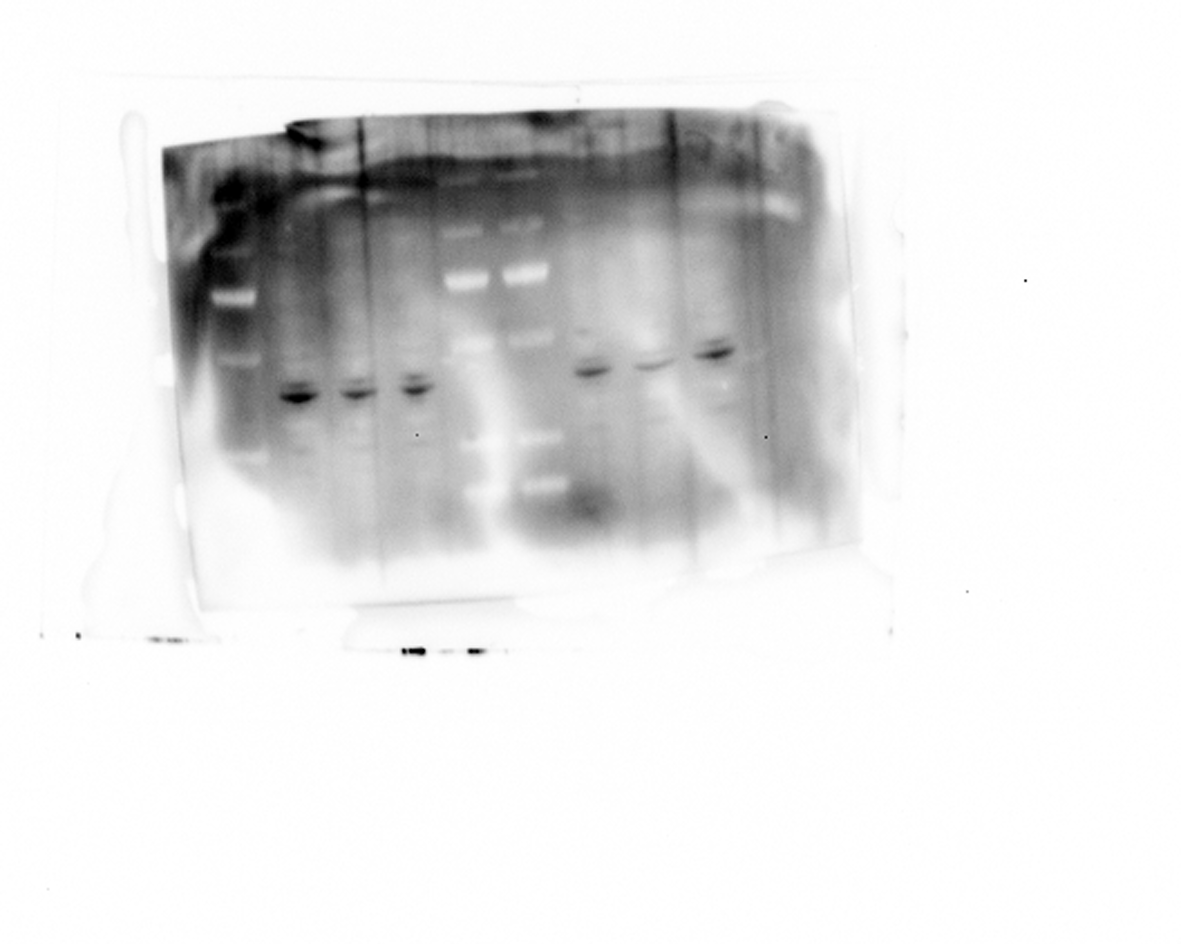

Supplement: Supplementary file 1 [file biomolecules-15-01493-s001.zip › H358_Experiment3_TTF1.tif]
